# A novel wide input range transformerless PV microinverter with natural power decoupling

**DOI:** 10.1038/s41598-025-15277-1

**Published:** 2025-08-22

**Authors:** Osama Elbaksawi, Youssef Nasr Ashour, Ahmed F. Abouzeid, Mahmoud Fawzi

**Affiliations:** Electrical Engineering Department, Faculty of Engineering, Port Said University, Port Said, Egypt

**Keywords:** Electrical and electronic engineering, Photovoltaics

## Abstract

In this paper, a novel wide range microinverter circuit that can interface with a single-phase grid and operates without a transformer is presented. The proposed topology uses six switches: two of those switches function at line frequency every half cycle while the other switches function at high switching frequency. In the proposed topology, the circuit functions in discontinuous conduction mode (DCM) across all possible operating conditions, ensuring high gain and minimal switching losses. A common connection between the PV panel and the grid exists, ensuring no common mode current. The proposed topology naturally decouples the power between the DC and AC sides without using an active power decoupling circuit. Passive power decoupling techniques implemented using a large electrolytic capacitor which is very well known to have low reliability is also not needed. Thus, the microinverter’s reliability is increased by using thin film capacitors. The analysis and verification of the proposed system are presented in this paper. Additionally, a standalone version of the presented circuit is verified experimentally through a fabricated prototype. In addition to the benefits of the presented circuit, both simulation and experimental data demonstrate that the circuit can operate without requiring a duty cycle constraint, offering significantly greater flexibility and a wider operating range.

## Introduction

Because of the benefits of being free of pollutants, renewable and sustainable, photovoltaic (PV) power is becoming more and more popular^1^. Three general categories can be distinguished: distributed, hybrid, and centralized systems. Centralized PV systems are large-scale solar farms that generate substantial amounts of electricity, typically feeding it directly into the grid. Distributed PV systems, on the other hand, generate electricity close to the point of consumption, such as on rooftops or in small local installations. Hybrid systems combine aspects of both centralized and distributed generation to optimize energy production and distribution. Among these, distributed PV systems offer particularly significant benefits. By producing electricity near where it is used, they minimize transmission losses and enhance the resilience of the power grid. This localized generation reduces the dependency on long-distance power transmission, lowering the risk of outages and increasing energy security. Furthermore, distributed photovoltaic systems are essential to the transition to renewable energy because they lower greenhouse gas emissions and lessen dependency on fossil fuels.

In distributed PV systems, a microinverter is required to integrate the generated direct current (DC) from the PV system into the alternating current (AC) form of the utility grids. A microinverter is a small inverter capable of handling low power suitable for distributed generation. Different topologies exist for these microinverters. Single-Stage Microinverters perform maximum power point tracking (MPPT) and conversion from DC to AC in a single phase^2^. They are simpler in design but might be less efficient in energy conversion under varying conditions. Two-Stage Microinverters separate the functions into two stages, MPPT and DC voltage regulation are handled by the first stage^3^. While DC-AC conversion is carried out by the second stage. This approach improves efficiency and flexibility, allowing for better optimization under different operating conditions. Galvanic isolation between the DC port and the AC port is achieved by isolated microinverters using transformers, enhancing safety and meeting regulatory requirements^4^. While adding to the cost and complexity. Non-Isolated Microinverters, in^5^, omit the transformer, leading to a simpler and more compact design, often resulting in higher efficiency and lower costs. Because of these advantages non-isolated microinverters are preferred for Distributed PV grid-integrated applications^6^.

However, because these inverter topologies lack the transformer. They are prone to common-mode leakage current^7,8^. Common-mode leakage current risks include reduced PV panel lifespan, the potential for electric shock, and the generation of electromagnetic interference^9–11^. There are two typical methods for preventing the common mode leakage current: (i) adding switches to single-phase full-bridge inverters, such as an H5 inverter^12^, H6 inverter^13^, or HERIC inverter^14^; (ii) using inverters equipped with an active virtual ground which work on the premise of isolating the PV panel from the AC power supply for a predetermined amount of time^11^. Nevertheless, each of these inverters must incorporate extra semiconductor components, which raises the level of control complexity and lowers efficiency^9^.

As an alternate method of removing common mode leakage current, a common connection between the PV panel and the AC supply is suggested^9^. In^15^, The microinverter circuit, which is built upon a buck-boost converter, uses one switch which function at high frequency and four switches which work at low frequency. A microinverter topology built from a Cuk converter that operates with all five switches at HF is shown in^16^. In^17^, A design for a microinverter using only four switches is suggested. According to^18^, output voltage with varying polarity can be generated with respect to a common ground by a microinverter design using just three switches operating at HF, but three inductors are also needed. It is necessary to state that every inverter discussed in^9,16,17^, and^18^ uses the continuous conduction mode (CCM), resulting in minimal voltage amplification factor.

The presence of a second-order harmonic signal at the input PV endpoint is another disadvantage of incorporating the PV system into the electrical grid with a single-phase inverter. This harmonic component causes a sizable voltage fluctuation across the PV panel which reduces the efficiency of maximum power point tracking (MPPT). The propagation of this harmonic component can be avoided using three techniques^19^: (i) passive methods (PPTs); (ii) active methods (APTs); (iii) control-oriented methods (CCTs).

PPTs are fairly simple to implement and don’t require any type of control. In these techniques, at the DC link or at the PV panel’s terminals, a sizable electrolytic capacitor is utilised to absorb the 2nd harmonic. These capacitors have a short life span and increased costs. According to the design requirements described in^20^, for unity power factor (UPF) operation, if the voltage ripple must be decreased to 5%. Then, 13 mF is the minimal capacitance that should be connected across the PV module. In^9,16–18^, electrolytic capacitors are used which reduces the overall reliability of these topologies.

APTs are typically involved in solving the aforementioned problems. These techniques usually depend on the presence of additional filters and switches that can be controlled to divert the ripple away from the PV side. This reduces the required capacitor value and increases the system overall lifetime. Different active power decoupling circuits (APDC) that require an additional connection across the PV terminals are presented in^20,21^. However, the system’s overall efficacy is decreased by the provided APDC’s requirement for additional HF switches and an inductor.

On the other hand, CCTs can be directly applied to two stage converters without the need for additional components. The primary idea is to create a control strategy that limits the 2nd harmonic ripple’s ability to propagate in the direction of the PV panel and forces it to confine at the DC link. But at the DC-link, this results in a deliberate rise in the harmonic pulsation. This in turn has a negative impact on the inverter’s output THD level. Two main ideas for these control methods are presented in literature. The first one depends on implementing dual-loop control mechanism modification. The technique makes use of a current mode control (CMC) system. As per^22^, it is possible to attain a lower value in the 2nd harmonic ripple at the input if the inner and outer-loop bandwidths are separated from 2nd harmonic frequency by at least half a decade. The fundamental concept is reducing the 2nd harmonic ripple component in the inner-loop controller’s reference current. But for load transients, the voltage-loop’s extremely low bandwidth (BW) results in a slow and poor system response. Consequently, Dynamic performance and second harmonic ripple reduction are traded off in this method. Shaping the output impedance is the foundation of the second strategy. The control modifies the Impedance at the output of the front-end DC-DC converter to increase the impedance of the second harmonic ripple with respect to the DC source. This approach suppresses ripples at the input, however the control results in a system with poor dynamic performance since the system has a high output impedance^23^. Both the output-impedance-shaping technique and the dual-loop control technique require maintaining the voltage-loop’s adequate bandwidth across the operation’s intended frequency range. Recently, better control methodologies have been shown to simultaneously boost the system response and decrease the 2ω − ripple at the DC input^24,25^. In the current or voltage loop, these control techniques employ resonant filters, such as band-pass filters (BPF) or notch-filters (NF).

Other efforts were presented to naturally achieve power separation in the primary inverting stage without the need to increase the bulk of the input electrolytic capacitor or introduce an additional APDC stage. In^5^, a Cuk-based microinverter that can naturally achieve power decoupling is presented. The circuit uses five high frequency (HF) switches which operates in CCM. The problem with instantaneous power mismatch is handled by the use of a capacitor at intermediate stage to operate the boost and buck stages independently^16^. Consequently, a PV module with a source input of 35 V can be connected to a 110 V AC supply using the architecture that has been given.

Different microinverter circuits which are capable of integrating the PV system into a 220 V AC grid supply using: (i) a Cuk and SEPIC-based high gain inductor-based circuit with six switches can be found in^26^; (ii) a coupled inductor, seven switches, and a boost flyback-based architecture are disclosed in^27^; (iii) a linked inductor-based microinverter that is based upon Cuk topology is created by one HF switch and four low frequency (LF) switches, as disclosed in^28^. However, the need for a sizable electrolytic capacitor or an extra APDC is the main drawback of the presented topologies. Moreover, topologies in^27,28^ increase the possibility of leakage current flow by connecting the neutral of the grid via a switch to the negative terminal of the PV module.

In^29^, the microinverter topology presented in^26^ is modified to include the second harmonic elimination controller for the boost and the buck-boost parts of the architecture. Furthermore, the presented topology operates in discontinues conduction mode (DCM) which increases the gain of the microinverter which enables a 35 V PV module to interface with a 220 V AC supply. The downside to this topology is that it’s not capable of producing reactive power. A buck-boost based, which is naturally able to perform power decoupling, is reported in^30^. The presented topology uses five HF switches and is intended to function in CCM. The buck-boost type of inverter can achieve different voltage levels for stand-alone as well as grid connected applications. Due to a shared connection between the grid’s neutral and the negative PV terminal, the topology also has very little leakage current. The demerit of the circuit is the gain changing for each half cycle makes the controller design as well as the parameters selection a difficult task.

In this paper, a novel non-isolated single-phase microinverter topology is proposed, aiming to enhance both control simplicity and system reliability. Building upon the strengths of the topology presented in^30^, the proposed circuit introduces a uniform voltage gain across both half-cycles of the grid, which significantly simplifies the control strategy and minimizes DC current injection into the grid. Additionally, the proposed topology offers a common ground connection and features inherent power decoupling capability, eliminating the need for extra active decoupling circuitry and reducing overall system complexity. The paper’s novel contribution can be summarized as follows:

- A novel microinverter topology that supports a wide range of input voltage with no floating interval for intermediate capacitance to ensure power decoupling is proposed.
- MATLAB/Simulink has been used to verify the suggested topology. The performance is evaluated for both grid connected and stand-alone applications.
- The proposed topology has been validated experimentally through a prototype.

The rest of the paper is organized as follows: Section II unveils the inverter topology. Section III introduces the specifications for the passive component design and operating range between different topologies. Section IV & V describes the circuit model in s-domain and the controller design. Section VI presents the simulation results for different operating conditions. Section VII presents the prototype components and experimental validation. Section VIII gives a comparative study between the presented work and the previously proposed Buck-Boost inverter.

## Proposed microinverter topology

**Fig. 1:**
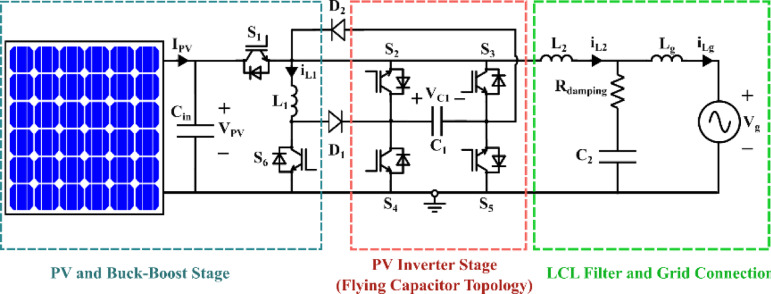
Diagram representation of the proposed microinverter circuit.

In this section, the proposed circuit operating principle will be analyzed. In addition, the constraints for the duty ratio for proper working of the presented circuit will be addressed. The topology of the suggested circuit is shown in Fig. 1 which contains six Isolated Gate Bipolar Transistor (IGBT) switches ($\:{S}_{1}$-$\:{S}_{6}$), two diodes ($\:{D}_{1}$ and $\:{D}_{2}$), inductor $\:\left({L}_{1}\right)$ attached to the PV input side, inductor $\:{\:(L}_{2})\:$connected to the output port, and inductor, $\:{\:(L}_{g})$ that interfaces the microinverter with the single-phase grid. The input voltage, output filter capacitor voltage, and grid voltage are denoted by $\:{V}_{pv}$, $\:{V}_{{C}_{2}}$, and $\:{V}_{g}$, respectively. To filter the 100 Hz grid power component and prevent voltage fluctuations across the input filter capacitance $\:{C}_{pv}$, an intermediate capacitor $\:{C}_{1}$, is used. An LCL output filter stage is made up of$\:{\:L}_{2}$, $\:{L}_{g}$, and $\:{C}_{2}$, as the output filter capacitor. The intrinsic resistances of the inductors $\:{\:L}_{1}$, $\:{\:L}_{2}$, and $\:{\:L}_{g}$ are$\:{\:r}_{1}$, $\:{\:r}_{2}$, and $\:{\:r}_{g}$, respectively.

A common connection between the PV side and the AC grid exist. This helps to eliminate common-mode (CM) leakage currents, which are a well-known issue in transformer-less inverter systems. These currents are primarily caused by parasitic capacitance between the PV panel and ground and can lead to electromagnetic interference (EMI), reduced efficiency, and safety concerns. By tying the PV negative terminal directly to the grid neutral (or ground), the voltage swing across the parasitic capacitance is minimized, thereby suppressing CM current generation. Inherent power decoupling is another merit of the design over traditional boost-cascaded H-bridge inverters.

### Operating principle

**Fig. 2:**
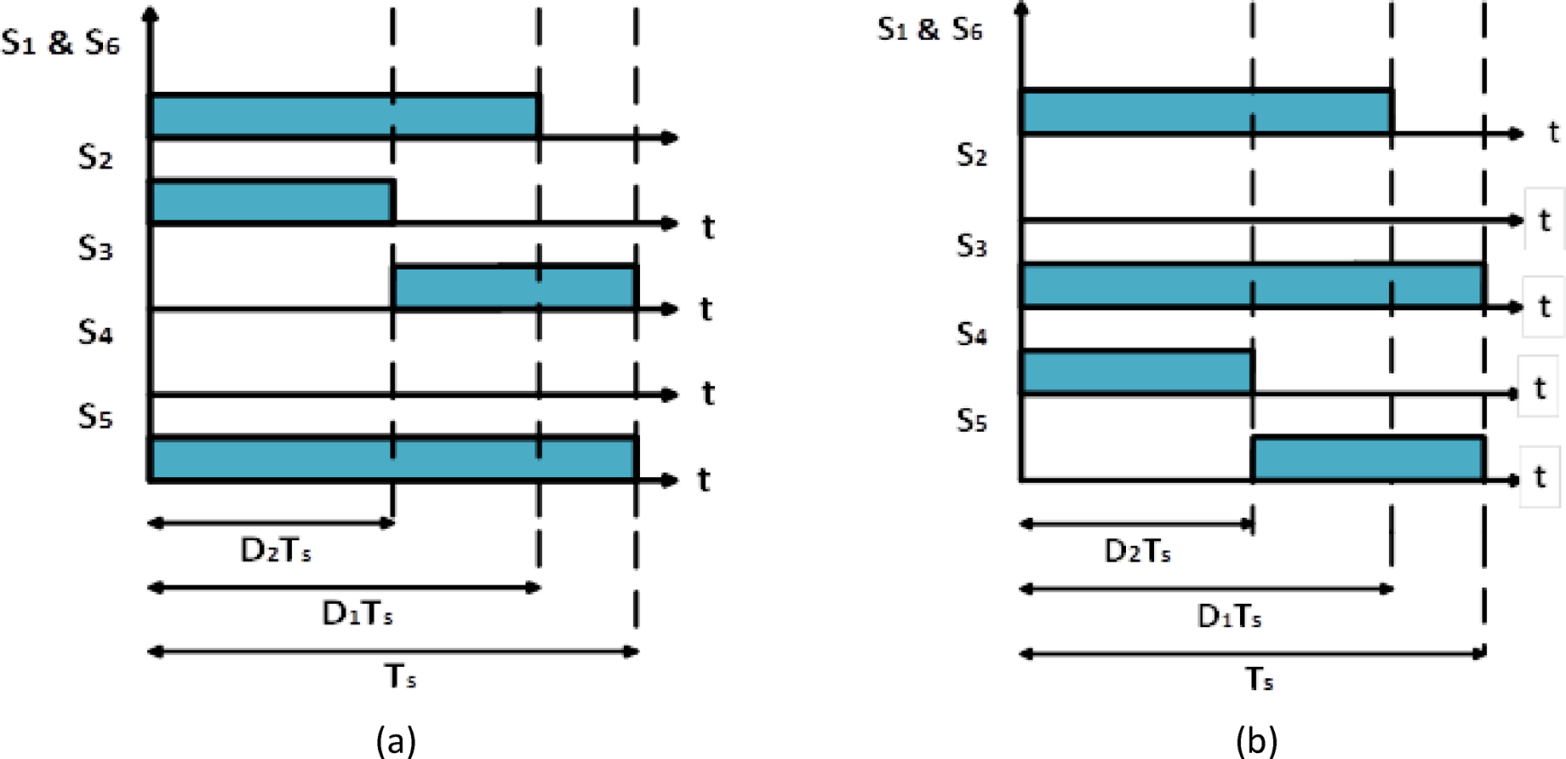
Switching pulses of the suggested circuit for: (a) positive and (b) negative output voltage polarities.

**Fig. 3:**
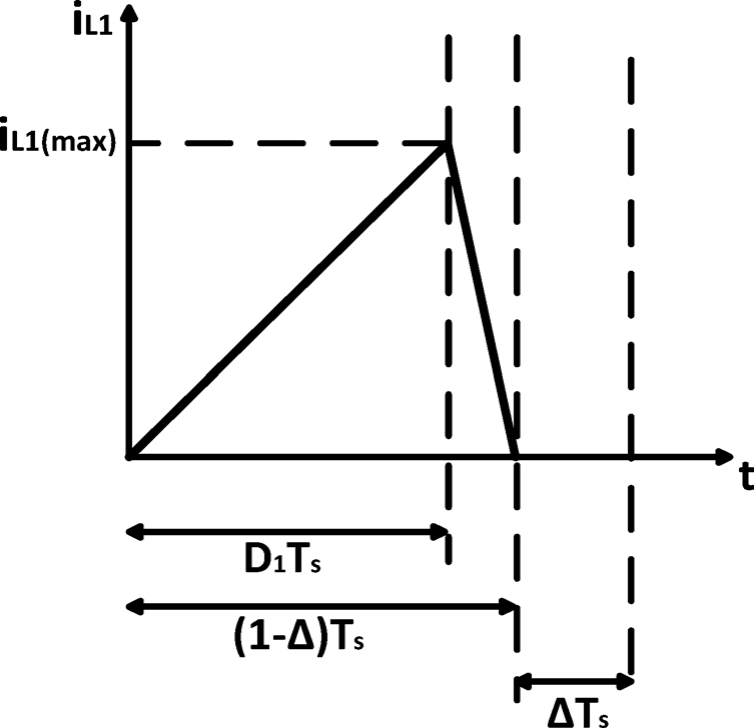
DCM operation of input inductor $\:{L}_{1}$.

The proposed circuit has two different modes of operation, i.e. positive and negative modes. In positive mode, the inverter stage operates as a typical buck converter to produce positive output voltage during the positive half cycle of the grid voltage. In negative mode, it operates as an inverted buck where the connections of capacitor $\:{C}_{1}$ are switched around during the negative half cycle of the grid voltage. Figure 2 illustrates the switching diagram for all power devices, where logic high (low) denotes the switches’ ON (OFF) states. If the switch combinations $\:{S}_{1}$ $\:{S}_{2}$ $\:{S}_{3}$ and $\:{S}_{2}$ $\:{S}_{4}$ $\:{S}_{5}$ are alternately turned on, it is possible to achieve both buck-boost and boost operation at the same time during a switching period. Another advantage of the suggested circuit is that the two stages’ voltage gains can be selected separately. Because the first stage is driven in DCM as shown in Fig. 3, the following advantages are provided:

- High Gain, converters operating in DCM generally exhibit a higher voltage gain compared to their continuous conduction mode (CCM) counterparts for the same duty ratio. This higher gain enhances the flexibility of the system, enabling it to interface with a broader range of grid voltage levels without requiring excessively high duty cycles. This is particularly beneficial in preventing saturation or operational limits when targeting specific AC output voltages.
- Low 2nd harmonic current component, DCM operation naturally results in a higher output impedance, especially at low frequencies. In the context of single-phase inverter applications, this is advantageous because it helps attenuate the second-order (100 Hz) power ripple from propagating back to the PV input. This supports the inherent power decoupling function of the proposed topology and reduces the reliance on large electrolytic capacitors.
- Facilitate controller design. DCM allows for faster dynamic response of the inductor current, which resets to zero each cycle. This makes the inductor current effectively instantaneous relative to the slower dynamics of the input capacitor voltage. As a result, the system dynamics can be approximated as a first-order system, significantly simplifying the control design and improving the regulation and tracking performance.

As will be shown when deriving the operational constraints, a significant advantage of the proposed circuit is that it eliminates the need for a floating interval across the intermediate capacitor, which increases flexibility in voltage gain design and enables higher efficiency to be attained for the circuit. Either way having an interval where the intermediate capacitor is completely isolated from the input and the output doesn’t change the operation and the equations of the system significantly. Thus, we will proceed to explain the working of the circuit taking into account the floating interval of the intermediate capacitor. On the other hand, the second stage is operated in CCM to ensure Lower grid current THD as well as the ability to control reactive power injection.

**Fig. 4:**
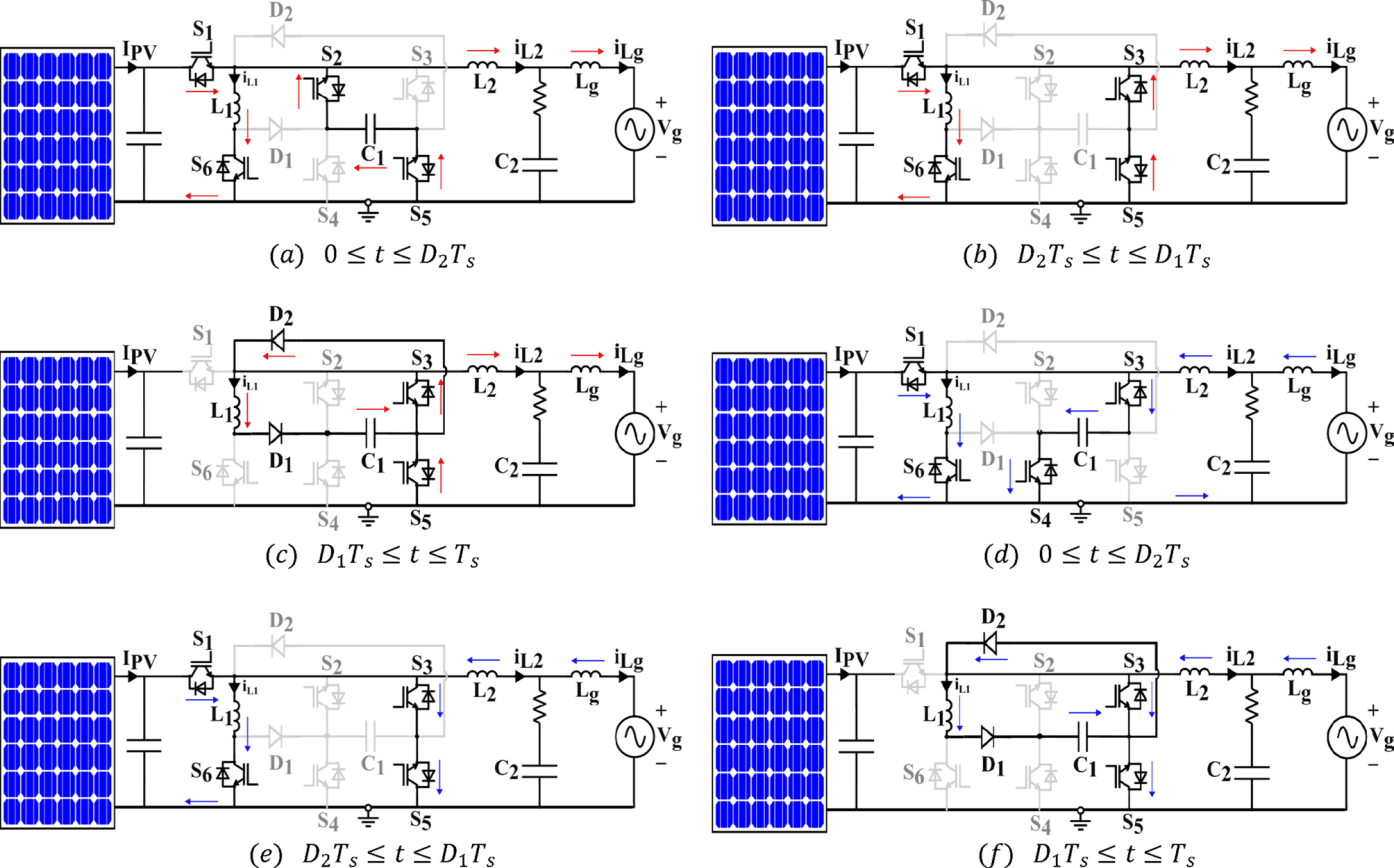
Equivalent diagram of the proposed circuit in varying operating conditions. Top: (a), (b) and (c) Positive mode. Bottom: (d), (e) and (f) Negative mode.

Figure 4 presents the equivalent circuits of the proposed topology in both modes at three different switching period intervals. The voltage across $\:{C}_{2}$, $\:{V}_{o}$, is regarded as the inverter’s output.

- Positive mode ($\:{V}_{o}>0$): Switches $\:{S}_{1}$, $\:{S}_{6}$, $\:{S}_{2}$, and $\:{S}_{5}$ are turned ON during the first part ($\:0\le\:t\le\:{D}_{2}{T}_{s}$) of the switching time interval $\:{T}_{s}$; the corresponding equivalent circuit is presented in Fig. 4a. The inductor $\:{L}_{1}$ current, $\:{i}_{{L}_{1}}$, flows as the energy moves from the input PV module $\:{V}_{pv}$. Similar to the conduction period of a buck converter, the energy in $\:{C}_{1}$ is moved to the output capacitor $\:{C}_{2}$. As depicted in the pertinent equivalent diagram of Fig. 4b, $\:{S}_{2}$ is turned OFF and $\:{S}_{3}$ is turned ON to start the floating interval of $\:{C}_{1}$ at $\:{t=D}_{2}{T}_{s}$. While current $\:{i}_{{L}_{1}}$ continues to rise with the same slope $\:\left(\frac{{V}_{pv}}{{L}_{1}}\right)$, $\:{i}_{{L}_{2}}$has a negative slope $\:\left(\frac{{-V}_{C2}}{{L}_{2}}\right).$ $\:{S}_{1}$ and $\:{S}_{6}$ are turned OFF at $\:{t=D}_{1}{T}_{s}$, but $\:{S}_{5}$ and $\:{S}_{3}$ remains ON, ending the floating interval of $\:{C}_{1}$. Figure 4c displays the same equivalent circuit. While $\:{i}_{{L}_{2}}$ keeps flowing with the same negative rate as in the preceding interval, stored energy in $\:{L}_{1}$ is transferred to $\:{C}_{1}$. In conclusion, the ON periods of the input and output buck-boost/Buck converters are represented by the intervals $\:\left(0<t<{D}_{1}{T}_{s}\right)$ and $\:(0<t<{D}_{2}{T}_{s})$, respectively.

The steady state average value of $\:{V}_{C1}$and $\:{V}_{o}$ is calculated using (1)-(2) considering the volt-sec balance in inductors $\:{L}_{1}$ and $\:{L}_{2}$. Therefore, the overall gain of the inverter in positive mode is given in (3).

1 $$\:\frac{{V}_{{C}_{1}}}{{V}_{pv}}=\frac{{D}_{1}}{{1-D}_{1}-\varDelta\:}$$

2 $$\:\frac{{V}_{o}}{{V}_{{C}_{1}}}={D}_{2}$$

3 $$\:\frac{{V}_{o}}{{V}_{pv}}=\frac{{D}_{1}{D}_{2}}{{1-D}_{1}-\varDelta\:}$$

Where $\:\varDelta\:\:$is the proportion of time where $\:{i}_{L1}$remains zero.

b) Negative Mode ($\:{V}_{o}<0$): In this operating mode, the switching logic is changed so that $\:{C}_{1}$ is connected in the other direction and a negative voltage applies across $\:{C}_{2}$. $\:{S}_{2}$ is completely OFF whereas $\:{S}_{3}$ remains ON the entire switching interval. As seen in Fig. 4d, switches $\:{S}_{1}$, $\:{S}_{3}$, and $\:{S}_{4}$, $\:{S}_{6}$ conduct during ($\:0\le\:t\le\:{D}_{2}{T}_{s}$). Since $\:{V}_{{C}_{1}}$ is kept at a voltage greater than $\:{V}_{o}$, $\:{i}_{{L}_{2}}$ grows in the opposite direction (negative slope), creating a negative voltage across $\:{C}_{2}$ as a result. The output buck stage’s ON time is determined by this interval. $\:{S}_{4}$ is turned OFF and $\:{S}_{5}$ is turned ON to start the floating interval of capacitor $\:{C}_{1},$ at $\:{t=D}_{2}{T}_{s}$. as in the positive mode. Once the energy kept in $\:{L}_{2}$ is moved to $\:{C}_{2}$, the slope of current $\:{i}_{{L}_{2}}$ reverses, but current $\:{i}_{{L}_{1}}$ continues to build up with the same rate as in the previous interval. Switches $\:{S}_{1}$ and $\:{S}_{6}$ are turned OFF at time $\:{t=D}_{1}{T}_{s}$ which finishes the floating interval of $\:{C}_{1}$ as well as the input buck-boost stage’s ON time. Energy stored in $\:{L}_{1}$ is moved to $\:{C}_{1}$ during the interval ($\:{D}_{1}{T}_{s}\le\:t\le\:{T}_{s}$) as$\:\:{i}_{{L}_{2}}$ flows through $\:{S}_{3}$ and $\:{S}_{5}$.

The average value of $\:{V}_{C1}$ and $\:{V}_{o}$ in a steady state is obtained in (4)-(5) using flux balance in $\:{L}_{1}$ and $\:{L}_{2}$. The gain of the circuit in negative mode is given by (6).

4 $$\:\frac{{V}_{{C}_{1}}}{{V}_{pv}}=\frac{{D}_{1}}{{1-D}_{1}-\varDelta\:}$$

5 $$\:\frac{{V}_{o}}{{V}_{{C}_{1}}}=-{D}_{2}$$

6 $$\:\frac{{V}_{o}}{{V}_{pv}}=\frac{-{D}_{1}{D}_{2}}{{1-D}_{1}-\varDelta\:}$$

c) Combined AC operation: By operating the circuit alternately in the two different modes described above, at 50 Hz frequency, it’s possible to create an output voltage that is closely like a sinusoidal voltage which is synchronized with the grid supply. The voltage gain in both modes is obtained by (7) and the voltage across the intermediate capacitor is given by (8).

7 $$\:\frac{{V}_{o}}{{V}_{pv}}=sgn\left({V}_{o}\right).\left(\frac{{D}_{1}.{D}_{2}}{{1-D}_{1}-\varDelta\:}\:\:\right)$$

8 $$\:{V}_{{C}_{1}}=\frac{{D}_{1}.{V}_{pv}}{{1-D}_{1}-\varDelta\:}=sgn\left({V}_{o}\right).\left(\frac{{V}_{o}}{{D}_{2}}\right)\:\:\:\:\:\:\:\:$$

Equation (8) demonstrates unequivocally the potential for regulating the voltage to be constrained to a specific range to separate control over $\:{V}_{pv}$ and $\:{V}_{o}$ which are independently controlled by $\:{D}_{1}$ and $\:{D}_{2}$, respectively. This serves as the foundation for the decoupled control strategy for the buck-boost and buck stages. In essence, the topology leverages the intermediate DC-link capacitor, which is maintained within a specific voltage range that satisfies the operational constraints of the converter. This regulation ensures that the two stages can operate independently: the first stage is dedicated to extracting maximum power from the PV source with a fixed or near-fixed duty cycle $\:{D}_{1}$​, while the second stage manages the grid-side current injection.

## Operational constraints

- Operation of the circuit when ($\:{D}_{1}\:$<$\:\:{D}_{2}$).

In this case, no floating interval exists for the decoupling capacitor. To investigate this effect, considering the equations of the inductors $\:{L}_{1}$ and $\:{L}_{2}$ expressed in (9)–(10). Then, applying the flux balance, it is found that Eq. (1) to (8) are still valid and the proposed circuit maintains its operation in terms of performance and its decoupling capability regardless the existence of a floating interval for the intermediate capacitor $\:{C}_{1}$. This provides the circuit a very wide range of operation which makes it suitable for different PV module current and voltage levels.

9 $$\:{{V}_{{L}_{1}}=L}_{1}\frac{d{i}_{{L}_{1}}}{dt}=\left\{\begin{array}{c}{V}_{pv}\:,\:\:\:0<t<\:{D}_{1}{T}_{s}\\\:-{V}_{{C}_{1}}\:\:,\:{D}_{1}{T}_{s}<t<\left(1-\varDelta\:\right)\:{T}_{s}\\\:0\:,\:\left(1-\varDelta\:\right){T}_{s}<t<\:{T}_{s}\end{array}\right.$$

10 $$\:{{V}_{{L}_{2}}=L}_{1}\frac{d{i}_{{L}_{2}}}{dt}=\left\{\begin{array}{c}{V}_{{C}_{1}}-{V}_{o}\:,\:\:\:0<t<\:{D}_{2}{T}_{s}\\\:-{V}_{o}\:\:,\:{D}_{2}{T}_{s}<t<\:{T}_{s}\end{array}\right.$$

b) Duty ratio constraint:

Despite the unconstrained operation advantage, with output voltage$\:{\:V}_{o}={V}_{g}\text{s}\text{i}\text{n}\left({\omega\:}_{g}t\right)$ and having a buck converter as an output stage some restriction on $\:{D}_{2}$ must be drawn for the inverter interfacing to the grid. The variation in $\:{D}_{2}$ can be found from (8) as given in (11). Thus, $\:{D}_{2}\left(t\right)$ shouldn’t exceed 1 as in (12). Therefore, condition (13) is the necessary criterion for proper operation of the circuit.

11 $$\:{D}_{2}\left(t\right)=\frac{{V}_{g}}{{V}_{{C}_{1}}\left(t\right)}\left|\text{sin}\left({\omega\:}_{g}t\right)\right|\:$$

12 $$\:{{D}_{2}}_{max}=\frac{{V}_{g}}{{{V}_{{C}_{1}}}_{min}}<1$$

13 $$\:{{V}_{{C}_{1}}}_{min}>{V}_{g}$$

### Hardware selection & range of operation

The hardware selection of the proposed circuit as well as the range of operations will be discussed in this section. The main circuit elements are selected to achieve proper operation.

- Hardware Selection.

Input inductor, $\:{L}_{1}$: As $\:{i}_{{L}_{1}}$is discontinuous, $\:{L}_{1}$ should be at least greater than twice the average current through it. Therefore:

14 $$\:{L}_{1\:}\le\:\frac{{V}_{pv}{V}_{{C}_{1}}{T}_{s}}{2{I}_{pv}\left({V}_{pv}+{V}_{{C}_{1}}\right)}$$

Interment capacitor, $\:{C}_{1}$: with a small capacitance value of $\:{C}_{1}$, the average voltage value, $\:{V}_{{C}_{1}}$, of the intermediate capacitor, $\:{C}_{1}$, is raised to permit a broader variation without polarity oscillation. Defining the grid current $\:{I}_{g}\left(t\right)\:$introduced into the grid supply at power factor angle $\:{\varnothing}$, as well as the grid voltage, $\:{V}_{g}\left(t\right)$, as:

15 $$\:{V}_{g}\left(t\right)={V}_{g}\text{s}\text{i}\text{n}\left({\omega\:}_{g}t\right)$$

16 $$\:{I}_{g}\left(t\right)={I}_{g}\text{s}\text{i}\text{n}({\omega\:}_{g}t-{\varnothing})$$

The instantaneous output power $\:{P}_{o}\left(t\right)$ is specified as:

17 $$\:P_{o} \left( t \right) = V_{g} \left( t \right) \times \:I_{g} \left( t \right) = \underbrace {{\frac{1}{2}V_{g} I_{g} {\text{cos}}\left( \emptyset \right)}}_{{P_{{dc}} }} + \underbrace {{\frac{1}{2}V_{g} I_{g} {\text{cos}}\left( {2\omega \:_{g} t - \emptyset } \right)}}_{{P_{{ac}} }}$$

The decoupling capacitor has the specific role to lessen the consequences of $\:{P}_{ac}$ on the voltages at the different parts of the inverter. To guarantee the single-phase inverter operates steadily, energy must be internally buffered.

18 $$\:{\int\:}_{\frac{-\pi\:}{4{\omega\:}_{g}}}^{\frac{\pi\:}{4{\omega\:}_{g}}}{P}_{ac}\:dt=\frac{{P}_{dc}}{{\omega\:}_{g}}\:\:$$

Thus, the decoupling capacitance needed to absorb the aforementioned energy is:

19 $$\:{C}_{1}=\frac{2{P}_{dc}}{{\omega\:}_{g}({{V}_{{C1}_{max}}}^{2}-{{V}_{{C1}_{min}}}^{2})}=\frac{{P}_{dc}}{{\omega\:}_{g}{V}_{{C}_{1}}\varDelta\:v\:}$$

Where $\:\varDelta\:v$ is the voltage variation. $\:{{V}_{{C}_{1}}}_{min}$choice is influenced by (10). The average voltages $\:{V}_{{C}_{1}}$ is determined by the selection of $\:{{V}_{{C}_{1}}}_{max}$and $\:{{V}_{{C}_{1}}}_{min}$.

A higher value of $\:{V}_{{C}_{1}}$ causes switching losses because it increases the voltage stress across all semiconductor devices. At a higher value of $\:{V}_{{C}_{1}}$, ripple in $\:{i}_{{L}_{1}}$ and $\:{i}_{{L}_{2}}$ likewise increases, resulting in higher conduction losses. According to (19), a greater value of $\:{V}_{{C}_{1}}$ reduces the decoupling capacitor’s size but increases the converter’s conduction losses and switching losses. Hence, $\:{V}_{{C}_{1}}$ is determined by striking a balance between size and converter loss.

Converter side inductor, $\:{L}_{2}$: the ripple expression for $\:{i}_{{L}_{2}}$, $\:\varDelta\:{i}_{{L}_{2}}$ is given by:

20 $$\:\varDelta\:{i}_{{L}_{2}}=\frac{{V}_{g}\left(1-{D}_{2}\right){T}_{s}}{{L}_{2}}$$

By deciding on $\:\varDelta\:{i}_{{L}_{2}}$, the value of $\:{L}_{2}$ is obtained according to:

21 $$\:{L}_{2}\ge\:\frac{{V}_{g}\left(1-{D}_{2}\right){T}_{s}}{\varDelta\:{i}_{{L}_{2}}}$$

Output capacitor, $\:{C}_{2}$: the output capacitor is chosen according to the reactive power consumption of the system as dictated by (22):

22 $$\:{C}_{2}=0.05{C}_{b}$$

Where $\:{C}_{b}$ is the base capacitance of the system and equals:

23 $$\:{C}_{b}=\frac{P}{{\omega\:}_{g}{{V}_{dc}}^{2}}$$

Where $\:{V}_{dc}$ is the DC link voltage.

Grid side inductance, $\:{L}_{\text{g}}$: the inductance of the grid is dictated by the attenuation of the harmonics injected by the converter side inductance as well as achieving a resonance frequency that is much less than the switching frequency.

24 $$\:{f}_{res}=\frac{1}{2\pi\:}\sqrt{\frac{{L}_{2}+{L}_{\text{g}}}{{L}_{2}{L}_{\text{g}}{C}_{2}}}\ll\:{f}_{sw}$$

Where $\:{f}_{res}$ and $\:{f}_{sw}$are the resonance and the switching frequencies respectively.

Damping resistance, $\:{R}_{damping}$: the damping of the LCL filter is required to dampen the resonance side effect from the filter. In this topology, passive damping technique has been chosen because of its simplicity, ease of implementation and cost reduction (i.e. no need to add another current sensor). The damping resistance is selected according to the expression:

25 $$\:{R}_{damping}=\frac{1}{3\times\:2\pi\:{f}_{res}{C}_{2}}$$

b) Range of operation.

The most remarkable advantage of having a buck-boost stage for the inverter is being capable of decreasing or raising the PV module voltage. Thus, enabling a broad spectrum of input PV Voltage and current. The unconstrained working of the suggested circuit gives an edge over the other existed microinverter circuits. Especially, the buck-boost inverter presented in^30^, where a limitation to this circuit is to have a floating interval for the intermediate capacitor in order to ensure power decoupling. This dictates that the duty cycle of the first stage $\:{{D}_{2}}^{{\prime\:}}$ must be greater than the duty cycle of the second stage $\:{D}_{1}$ which can be represented ad the following constraint:

26 $$\:\frac{{V}_{{C}_{1}}}{{V}_{{C}_{1}}+{V}_{pv}}>\frac{{V}_{grid}}{{V}_{{C}_{1}}}$$

Other constraints exist for the proper operation of the circuit as indicated by the inequalities:

27 $$\:{V}_{{C}_{1}}>{V}_{grid}$$

28 $$\:\left(1-{D}_{1max}-{D}_{2}\right){T}_{s}\frac{{V}_{{C}_{1}}}{{L}_{1}}>{I}_{2}+{D}_{1max}{T}_{s}\frac{{V}_{{C}_{1}}+{V}_{pv}}{{2L}_{2}}$$

**Fig. 5:**
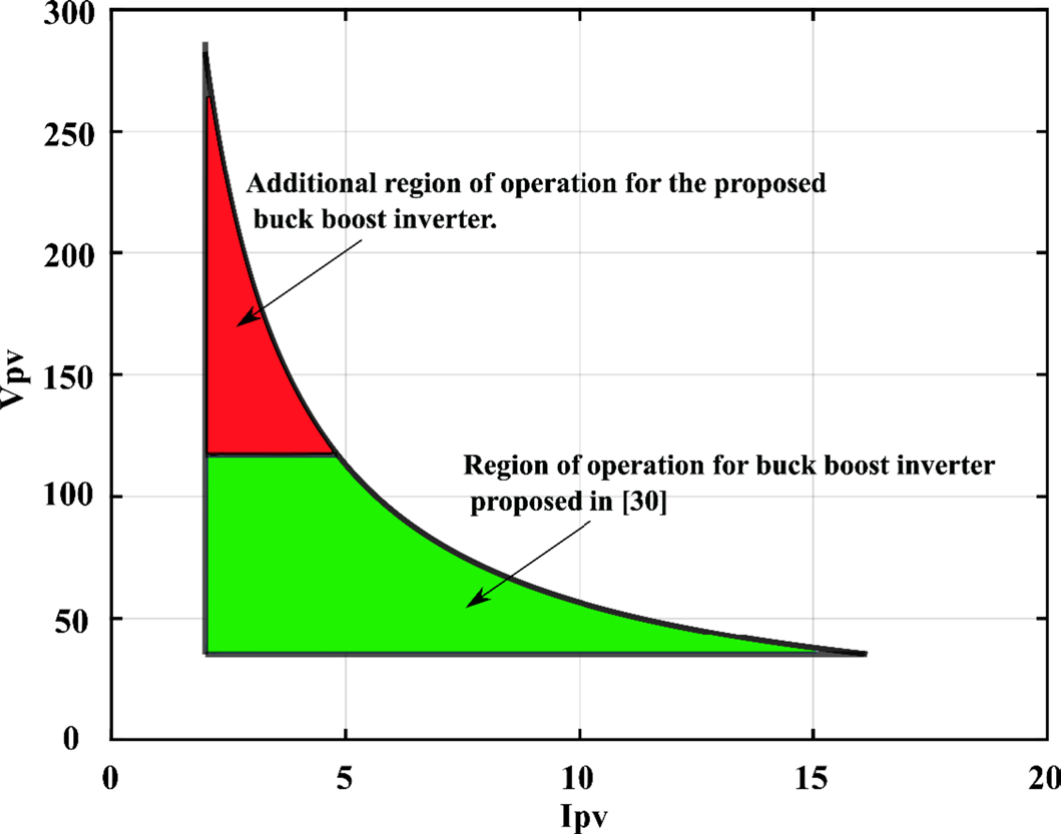
Range of operation for the proposed topology vs. the topology proposed in^30^.

These constraints limit the range of operation of the circuit. For a fair comparison with the proposed microinverter topology, a practical limit for the input PV module voltage and current is set as 35 V and 2 A. Also, the ripple is limited to a maximum of 20 V, the different constraints for both circuits are taken into account and the feasible region of operation is presented in Fig. 5. The Buck-Boost inverter input ranges from 35 V to 117 V. This estimation agrees with the range of operation proposed in^30^ (40–100 V). However, the proposed topology can accommodate higher input voltage (up to 270 V), thus it has a wider range of operation and more versatile.

## Mathematical model

In this section, the model for the circuit is derived. The inverter is divided into two parts: a buck-boost stage and a buck stage by the intermediate capacitor $\:{C}_{1}$. A controller with a smaller bandwidth and a large capacitor is used to manage $\:{V}_{{C}_{1}}$. The capacitor is used as an approximate equivalent for a voltage source when developing current controllers because of its slower dynamics.

### Buck-boost stage

Since $\:{i}_{{L}_{1}}$ is discontinuous, it cannot be regarded as a state^31^. This reduces the system to a first order system, which minimizes the difficulty of the buck-boost stage controller design. $\:{V}_{pv}$ is the only state in this stage.

29 $$\:{C}_{in}\frac{d{V}_{pv}}{dt}=\left\{\begin{array}{c}{I}_{pv}-{i}_{{L}_{1}}\:\:,\:\:\:0<t<\:{D}_{1}{T}_{s}\\\:{I}_{pv}\:\:\:\:\:\:\:\:\:\:,\:{D}_{1}{T}_{s}<t<\:\:{T}_{s}\end{array}\right.$$

Using state-space averaging, the mean voltage across the PV module over a switching cycle $\:{T}_{s}$ is given by:

30 $$\:{C}_{in}\frac{d{V}_{pv}}{dt}={I}_{pv}-\frac{1}{{T}_{s}}{\int\:}_{0}^{{D}_{1}{T}_{s}}{i}_{{L}_{1}}\:dt\:$$

Since $\:{i}_{{L}_{1}}$ is discontinuous the average current over a switching period is defined as:

31 $$\:\frac{1}{{T}_{s}}{\int\:}_{0}^{{D}_{1}{T}_{s}}{i}_{{L}_{1}}\:dt=\frac{{V}_{pv}{{D}_{1}}^{2}{T}_{s}}{2{L}_{1\:}}\:$$

Substituting (31) into (30):

32 $$\:{C}_{in}\frac{d{V}_{pv}}{dt}={I}_{pv}-\frac{{V}_{pv}{{D}_{1}}^{2}{T}_{s}}{2{L}_{1\:}}\:\:$$

By applying a small perturbation to (32), it is found:

33 $$\:{C}_{in}\frac{d\stackrel{\sim}{{V}_{pv}}}{dt}=\stackrel{\sim}{{I}_{pv}}-\frac{{\stackrel{-}{{D}_{1}}}^{2}{T}_{s}}{2{L}_{1\:}}\stackrel{\sim}{{V}_{pv}}-\frac{\stackrel{-}{{V}_{pv}}{T}_{s}\stackrel{-}{{D}_{1}}}{{L}_{1\:}}\stackrel{\sim}{{D}_{1}}\:\:$$

Since $\:\stackrel{\sim}{{I}_{pv}}$ depends on irradiance and temperature, then it can be considered a low-frequency disturbance that can be rejected easily by the controller. Thus, the s-domain transfer function for the buck-boost stage can be written as:

34 $$\:{G}_{1}\left(s\right)=\frac{\stackrel{\sim}{{V}_{pv}}}{\stackrel{\sim}{{D}_{1}}\:}=\frac{-\frac{\stackrel{-}{{D}_{1}}\times\:\stackrel{-}{{V}_{pv}}{T}_{s}}{{L}_{1\:}}}{{C}_{in}s+\frac{{\stackrel{-}{{D}_{1}}}^{2}{T}_{s}}{2{L}_{1\:}}}$$

Where $\:\stackrel{-}{{D}_{1}}$and $\:\stackrel{-}{{V}_{pv}}$ are the steady state values of the duty cycle and PV module voltage respectively. Substituting the steady-state values at STC as well as the relevant system and inverter parameters:

35 $$\:{G}_{1}\left(s\right)=\frac{\stackrel{\sim}{{V}_{pv}}}{\stackrel{\sim}{{D}_{1}}\:}=\frac{-20.667}{{10}^{-4}s+0.1281}$$

**Fig. 6:**
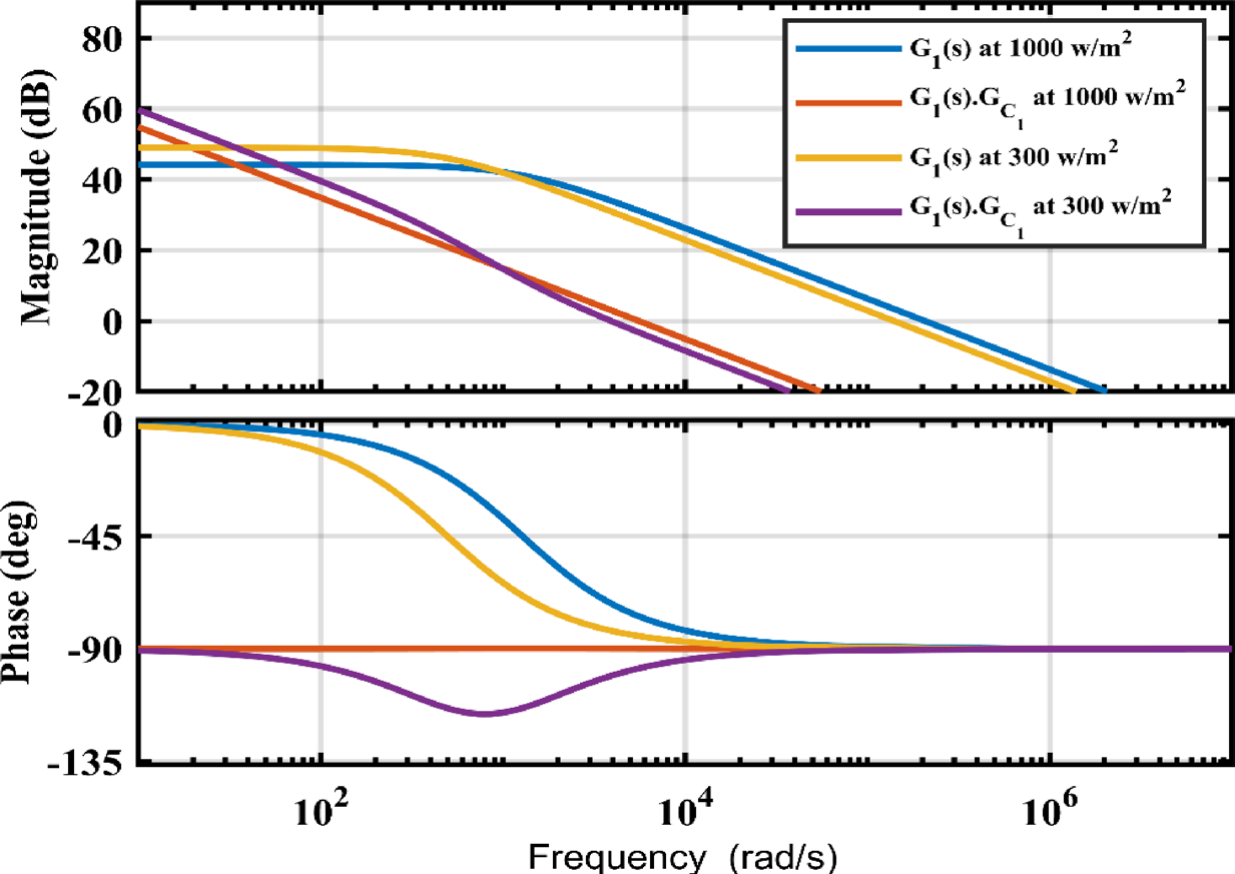
Frequency plot of input buck boost stage at 1000 $\:w/{m}^{2}$ 300 $\:w/{m}^{2}$ and and 25 °C.

From (35) it can be shown that $\:{G}_{1}\left(s\right)$ is a system of first order and $\:{G}_{{c}_{1}}\left(s\right)$ is the input voltage controller. A PI controller with $\:{K}_{p}\:=\:0.02691\:$and $\:{K}_{i}\:\:=\:34.319\:{s}^{-1}\:$is used to implement the controller $\:{G}_{{c}_{1}}\left(s\right)$, and the equivalent Gain Cross over Frequency (GCF) of the compensated system is 898 Hz. The operational point and the transfer function, $\:{G}_{1}\left(s\right)$, both shift when the irradiance level varies. It is shown that when the irradiance level shifts from 300 to 1000$\:\:w/{m}^{2}$, the GCF fluctuates between 624 and 898 Hz as shown in Fig. 6.

### Buck stage

In this stage, three states exist. The system’s input and state variables are:

36 $$\:\varvec{X}={\left[\:{i}_{{L}_{2}}\:\:\:{i}_{g}\:{\:V}_{{C}_{2}}\:\right]}^{T}\text{a}\text{n}\text{d}\:\varvec{u}={\left[\:\:\:{V}_{inv}\:\:\:{V}_{g}\:\right]}^{T}$$

Where $\:{i}_{\text{g}}\:$is the controlled variable and $\:{V}_{inv}$ is the average inverter output.

37 $$\:{V}_{inv}={{D}_{2}\:V}_{{C}_{1}}$$

The average state space model can be written in general terms as:

38 $$\:\frac{d}{dt}\varvec{X}=\varvec{A}\varvec{X}+\varvec{B}\varvec{u}$$

$$\:\varvec{Y}=\varvec{C}\varvec{X}+\varvec{D}\varvec{u}$$

The average state space equation for the buck stage can be obtained as in (38):

39 $$\:\frac{d}{dt}\left[\begin{array}{c}{i}_{{L}_{2}}\\\:{i}_{g}\\\:{\:V}_{{C}_{2}}\end{array}\right]=\left[\begin{array}{ccc}\frac{-{r}_{2}-R}{{L}_{2}}&\:\frac{R}{{L}_{2}}&\:-\frac{1}{{L}_{2}}\\\:\frac{R}{{L}_{g}}&\:\frac{-{r}_{g}-R}{{L}_{g}}&\:\frac{1}{{L}_{g}}\\\:\frac{1}{{C}_{2}}&\:-\frac{1}{{C}_{2}}&\:0\end{array}\right]\left[\begin{array}{c}{i}_{{L}_{2}}\\\:{i}_{g}\\\:{\:V}_{{C}_{2}}\end{array}\right]+\left[\begin{array}{cc}\frac{1}{{L}_{2}}&\:0\\\:0&\:-\frac{1}{{L}_{g}}\\\:0&\:0\end{array}\right]\left[\begin{array}{c}{{D}_{2}\:V}_{{C}_{1}}\\\:{V}_{g}\end{array}\right]$$

$$\:{i}_{g}=\left[\:0\:\:1\:0\right]\left[\begin{array}{c}{i}_{{L}_{2}}\\\:{i}_{g}\\\:{\:V}_{{C}_{2}}\end{array}\right]+\left[\:0\:\:\:0\:\right]\left[\begin{array}{c}{{D}_{2}\:V}_{{c}_{1}}\\\:{V}_{g}\end{array}\right]$$

Therefore, it can be found that:

40 $$\:\varvec{A}=\left[\begin{array}{ccc}\frac{-{r}_{2}-R}{{L}_{2}}&\:\frac{R}{{L}_{2}}&\:-\frac{1}{{L}_{2}}\\\:\frac{R}{{L}_{g}}&\:\frac{-{r}_{g}-R}{{L}_{g}}&\:\frac{1}{{L}_{g}}\\\:\frac{1}{{C}_{2}}&\:-\frac{1}{{C}_{2}}&\:0\end{array}\right],\:\:\:\:\:\:\:\varvec{B}=\left[\begin{array}{cc}\frac{1}{{L}_{2}}&\:0\\\:0&\:-\frac{1}{{L}_{g}}\\\:0&\:0\end{array}\right],\varvec{C}=\left[\:0\:\:1\:0\right],\:\:\:\:\:\:\:\varvec{D}=\left[\:0\:\:\:0\:\right].$$

The grid voltage is considered a disturbance and introduces error in the phase and magnitude of the commanded current that could be rejected by a high gain properly tuned proportional resonant (PR) controller. Thus, it will be eliminated from the above model. The s-domain transfer function relating the grid current $\:{i}_{g}$ and the duty cycle $\:{D}_{2}$ can be found as:

41 $$\:{G}_{2}\left(s\right)=\frac{\stackrel{\sim}{{i}_{g}}}{\stackrel{\sim}{{D}_{2}}\:}{=C(sI-A)}^{-1}B+D=\frac{{\:{V}_{{C}_{1}}}^{*}({C}_{2}Rs+1)}{{a}_{3}{s}^{3}+{a}_{2}{s}^{2}+{a}_{1}s}$$

Where $\:{\:{V}_{{C}_{1}}}^{*}$(or$\:{{\:V}_{dc}}^{\text{*}})$ is the reference DC link voltage and $\:\:{r}_{2}$ and $\:{r}_{g}$have been omitted because they have a very small value and the values of the constants:

42 $$\:{a}_{3}={L}_{g}{L}_{2}{C}_{2},\:\:\:\:{a}_{2}=R{C}_{2}{(L}_{g}+{L}_{2}),\:{\:\:\:a}_{1}={(L}_{g}+{L}_{2})$$

By substituting the system and inverter parameters into (41), the $\:{G}_{2}\left(s\right)$ can be given as:

43 $$\:{G}_{2}\left(s\right)=\frac{\stackrel{\sim}{{i}_{g}}}{\stackrel{\sim}{{D}_{2}}\:}=\frac{0.0017s+200}{1.594{\times\:10}^{-12}{s}^{3}+1.168{\times\:10}^{-8}{s}^{2}+0.001375s}$$

**Fig. 7:**
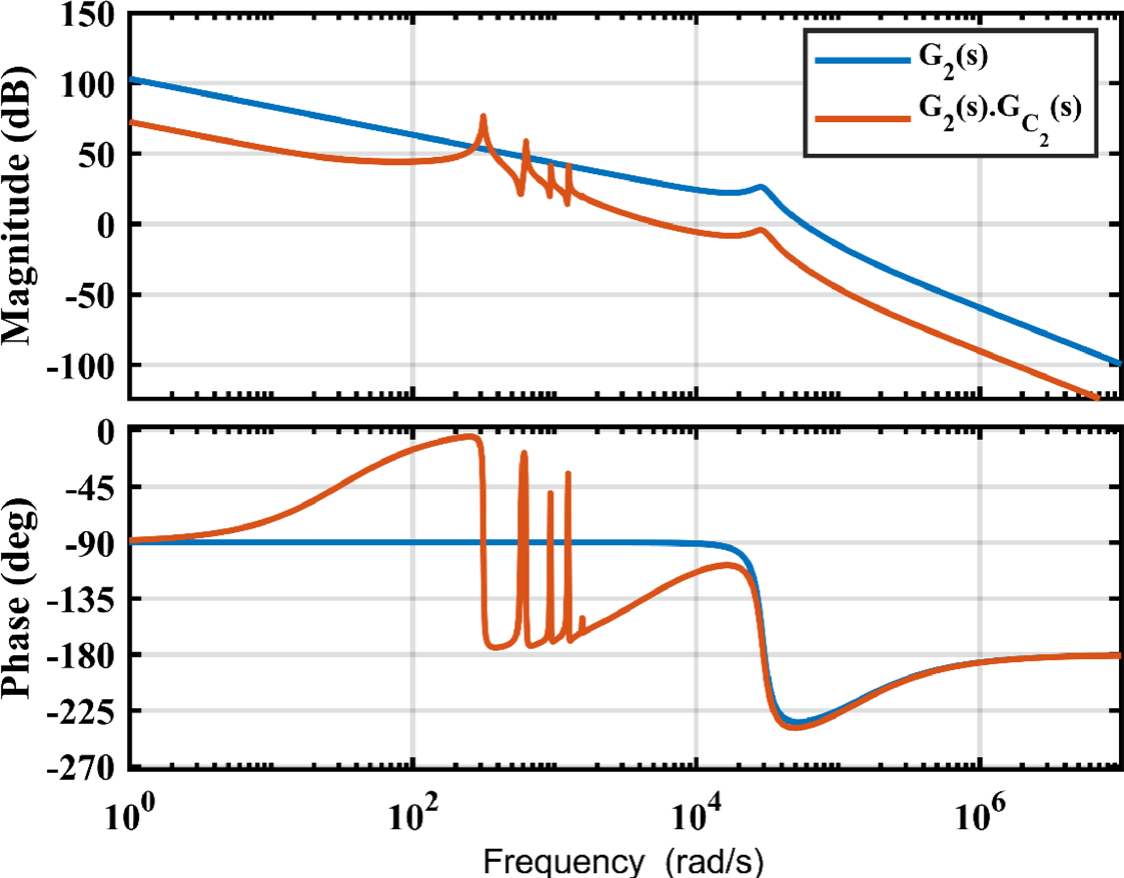
Frequency plot of the output buck stage.

A proportional resonant controller and harmonic compensator are used to regulate the output grid current and reduce the harmonics in the system. The frequency response of the open loop uncompensated system $\:{G}_{2}\left(s\right)$ and the compensated system $\:{G}_{{c}_{2}}\left(s\right)\text{*}{G}_{2}\left(s\right)$ is shown in Fig. 7. The compensated system is shown to have approximately a phase margin of $\:55.5^\circ\:$ and a gain margin of $\:4.75\:$dB as well as a bandwidth frequency of $\:5330\:rad/s$. The system has a high gain at the grid frequency and its harmonics to ensure proper reference tracking as well as disturbance rejection of the associated signals. The values used for the PR harmonic compensator are shown in Table I.

**Table 1:** Proportional resonant compensator parameters.

Frequency	$\:{K}_{p}$	$\:{K}_{i}$
$\:50\:Hz$	0.02925	14.38
$\:100\:Hz$	-	3.636
$\:150\:Hz$	-	0.7272
$\:200\:Hz$	-	0.992
$\:250\:Hz$	-	0.02067

**Fig. 8:**
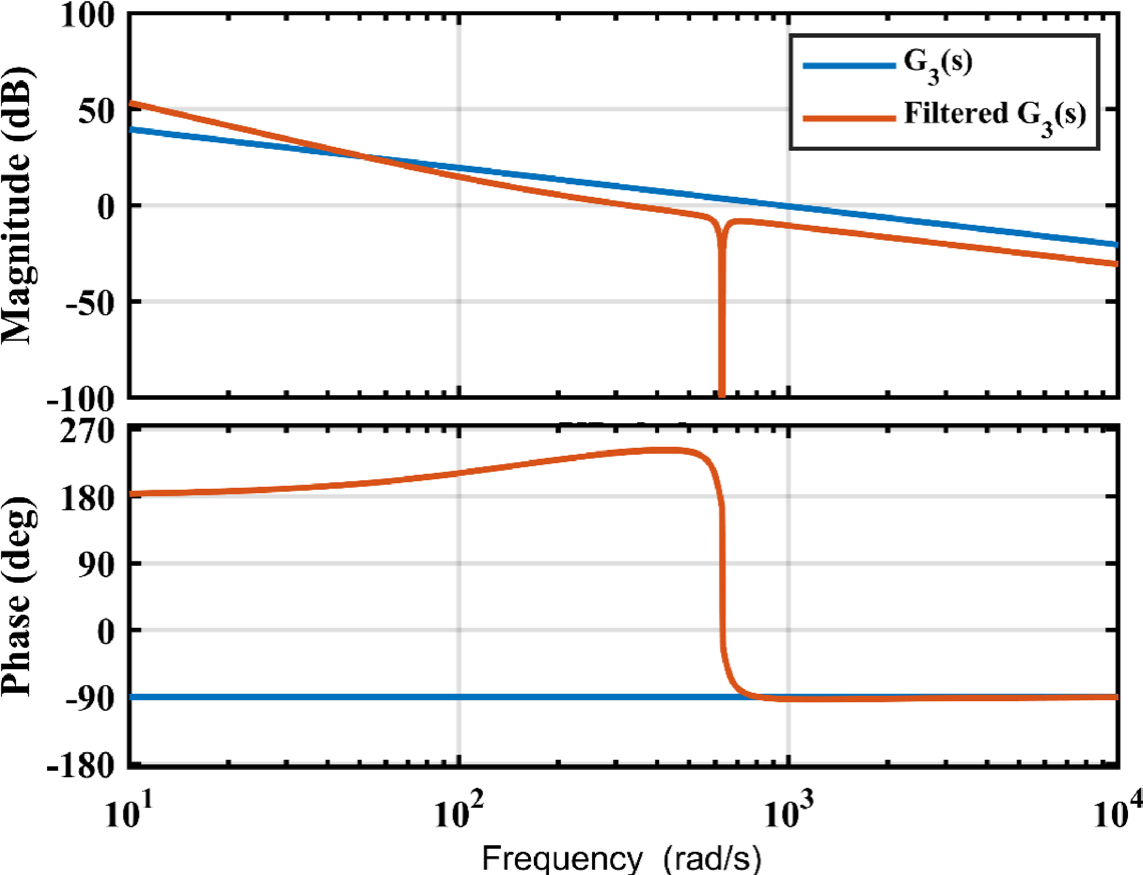
Frequency plot of the DC link voltage with notch filter.

In the current control loop, $\:{\:{V}_{{C}_{1}}}^{*}$determines the current amplitude. A PI controller is responsible for keeping $\:{\:V}_{{C}_{1}}$ at a predetermined voltage that complies with (10). The reference amplitude, $\:{{\:I}_{g}}^{\text{*}}$, is produced at the DC link voltage controller output. The reflection of pulsating output power is additionally filtered out using a notch filter calibrated at 2$\:{\omega\:}_{g}$. The plant transfer operation along with the open loop compensated system is presented in Fig. 8.

The s-domain transfer function for the DC link voltage can be found as:

44 $$\:{G}_{3}\left(s\right)=\frac{\stackrel{\sim}{{V}_{DC}}}{\stackrel{\sim}{{I}_{g}}\:}=\frac{-{V}_{g}}{{2{{\:V}_{{C}_{1}}}^{\text{*}}C}_{in}s}$$

For proper operation of the cascaded control paradigm, the inner current loop bandwidth has to be at least five times the outer DC link voltage controller bandwidth, which is realized in the compensated system where the PI controller parameters are chosen as:

45 $$\:{K}_{p}=0.31158,\:\:\:\:\:\:\:{K}_{i}=49.447$$

The notch filter in a way to enable proper filtration of the 2nd harmonic ripple in the feedback path as well as achieving a high bandwidth. The center frequency $\:{f}_{c}=100\:Hz$ and the damping ratio $\:{\upbeta\:}=20{\uppi\:}.$ These parameters dictate the bandwidth to a value of $\:620\:rad/s$, a phase margin of $\:60^\circ\:,$ and a gain margin of $\:18.3\:dB$. This ensures fast transient response and sufficient stability margins.

## Control method

In this section, the control methodology for proper operation is explained in detail. The controller’s goal is to extract MPP current from the PV source while supplying almost a pure sinusoidal current to the grid.

### Grid connected system

**Fig. 9:**
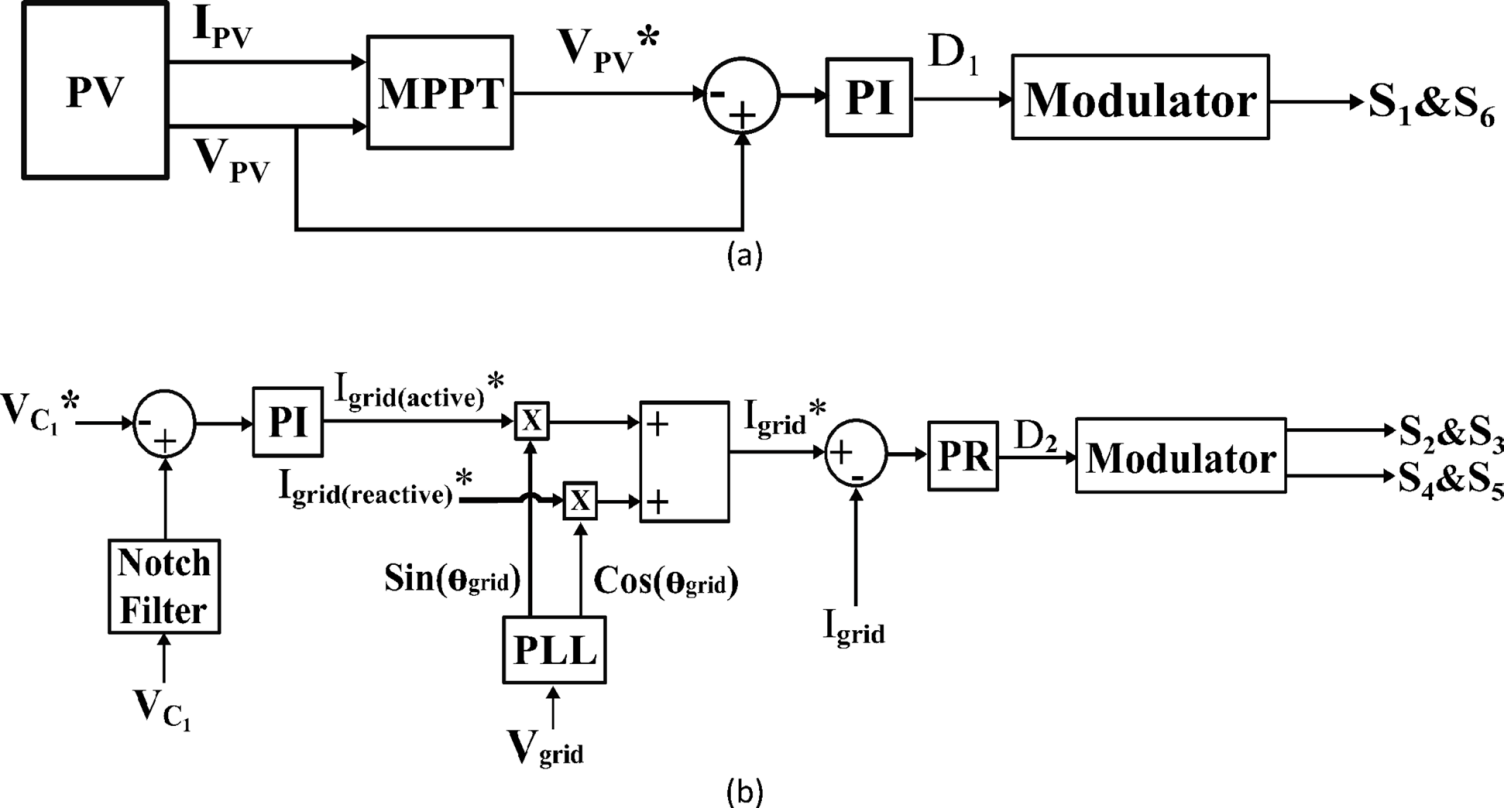
The illustration of the control setup for: (a) MPPT and (b) grid current control.

Figure 9a presents the input voltage controller for the buck-boost side where a PI controller is used to maintain the voltage across the PV module. The PI controller produces $\:{D}_{1}$ by processing the error between the reference and feedback signal, $\:{D}_{1}$ is then used to derive the switching logic for both $\:{S}_{1}$ and $\:{S}_{6}$. Using an incremental conductance-based MPPT algorithm^32^, the solar PV panel’s maximum available power can be extracted.

Figure 9b demonstrates the output current controller for the grid inverter side. By using a notch filter adjusted to 100 Hz, the average voltage, $\:{\:V}_{{C}_{1}}$, across $\:{C}_{1}$ is computed. A PI controller processes the error to produce the reference peak value of the output grid current, $\:{I}_{g}$*, by comparing the average DC voltage with a specified reference, $\:{\:V}_{{C}_{1}}$*.

A sinusoidal output current is achieved by using the Phase-Locked Loop (PLL) which is synchronized with the grid voltage. Then, the sinusoidal current reference is checked with the feedback grid current. The difference is processed through a Proportional Resonant (PR) controller tuned at 50 Hz to accomplish zero steady-state error for sinusoidal signals. The obtained duty cycle $\:{D}_{2}$ of the buck converter is then modulated to produce the switching logic for $\:{S}_{2}$, $\:{S}_{3}$, $\:{S}_{4}$, and $\:{S}_{5}$.

### Standalone system

**Fig. 10:**
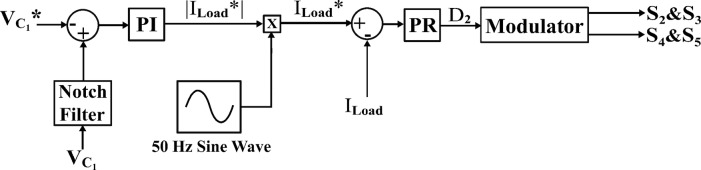
The illustration of the control setup for standalone applications.

For standalone applications, the control methodology depends on the control objective whether it is to supply regulated voltage to a load or to provide maximum power. The latter case is adopted in this paper. $\:{D}_{1}$ is generated using the same control loop in Fig. 9a. simply the first stage controls and outputs the duty cycle to get the highest power from the solar module. Also, the 2nd stage controller’s main objective remains the same. That is to regulate the DC link voltage at a predetermined level. The only discrepancy could be pointed out is the fact that there is no grid to synchronize the current with, so a 50 Hz sine wave template is fed for the controller as shown in Fig. 10.

## Simulation results

### Grid connected system

With MATLAB Simulink, the inverter is simulated. Table 2 contains a list of the system & inverter parameters. Three levels of solar insolation are used to assess the MPP’s functionality.

**Table 2:** The system & inverter parameters.

Parameters	Values
System Parameters	
$\:{V}_{mpp}$ and $\:{{V}_{oc}}^{1}$ at STC^2^	50 V and 60 V
$\:{I}_{mpp}$ and $\:{{I}_{sc}}^{3}$at STC	7.65 A and 8.42 A
MPP Power, $\:{P}_{mpp}$at STC	384 W
Grid Voltage (RMS), $\:{V}_{g}$	120 V
Grid frequency, $\:{f}_{g}$	50 HZ
Switching frequency, $\:{f}_{sw}$	20 KHZ
Inverter Parameters	
$\:{C}_{in}$	100 µF
$\:{L}_{1\:},\:{r}_{1}$	60 µH, 15 mΩ
$\:{C}_{1}$	450 µF
$\:{L}_{2},\:{\:r}_{2}$	375 µH, 15 mΩ
$\:{C}_{2}$	4.25 µF
$\:{L}_{g},\:{r}_{g}$	1 mH, 15 mΩ
$\:{R}_{damping}$	2 Ω
$\:{{V}_{dc}}^{\text{*}}$	200 V

1$\:\:{V}_{oc}$ Open circuit voltage of the PV module.

2 STC: Standard test condition (Solar insolation = 1 kW/m2, Temp.=25◦C).

3 $\:{I}_{sc}$: Short circuit current of the PV module.

**Fig. 11:**
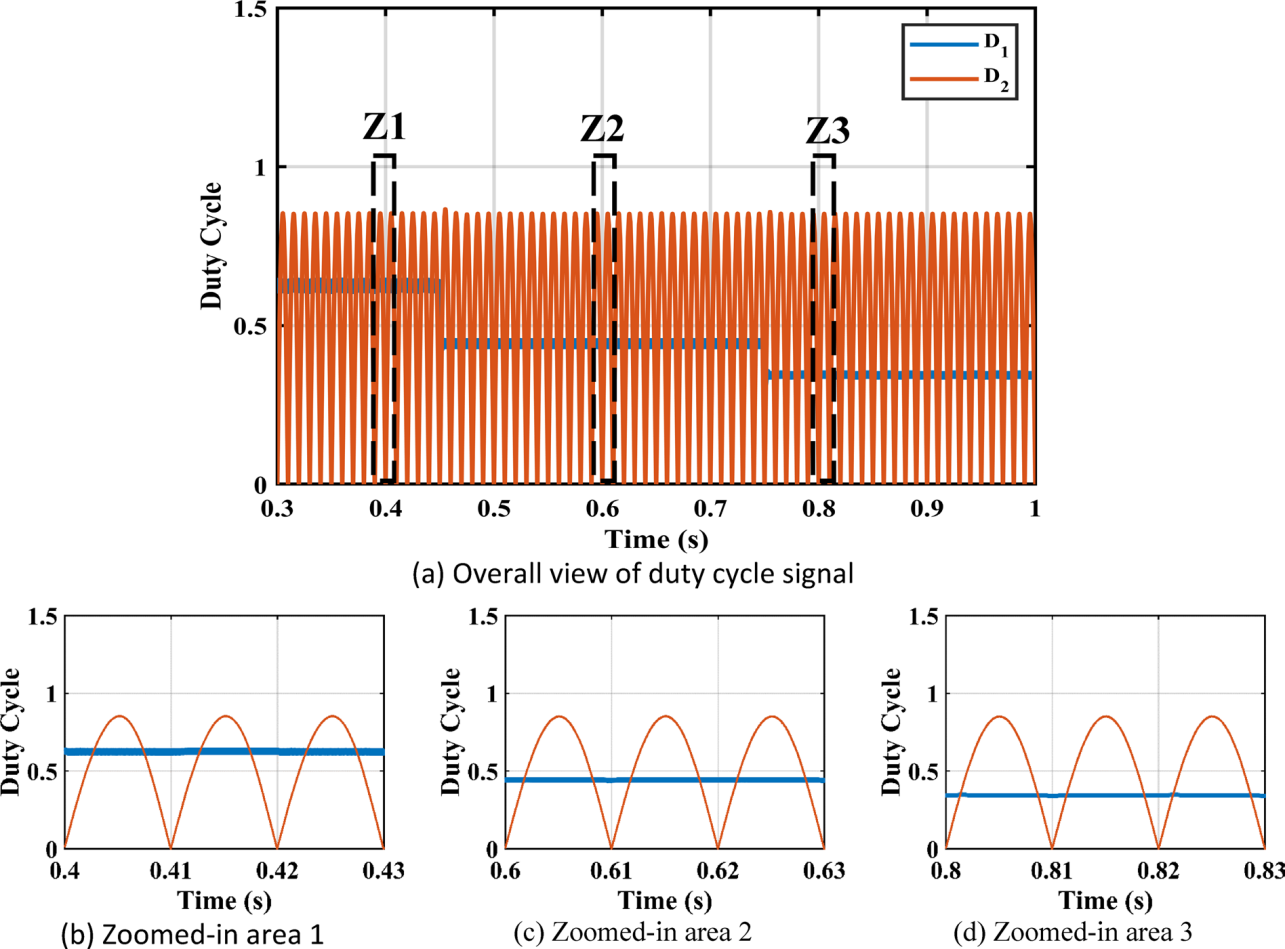
Duty ratio variations for the input $\:{D}_{1}$ and the output stage $\:{D}_{2}.$.

**Fig. 12:**
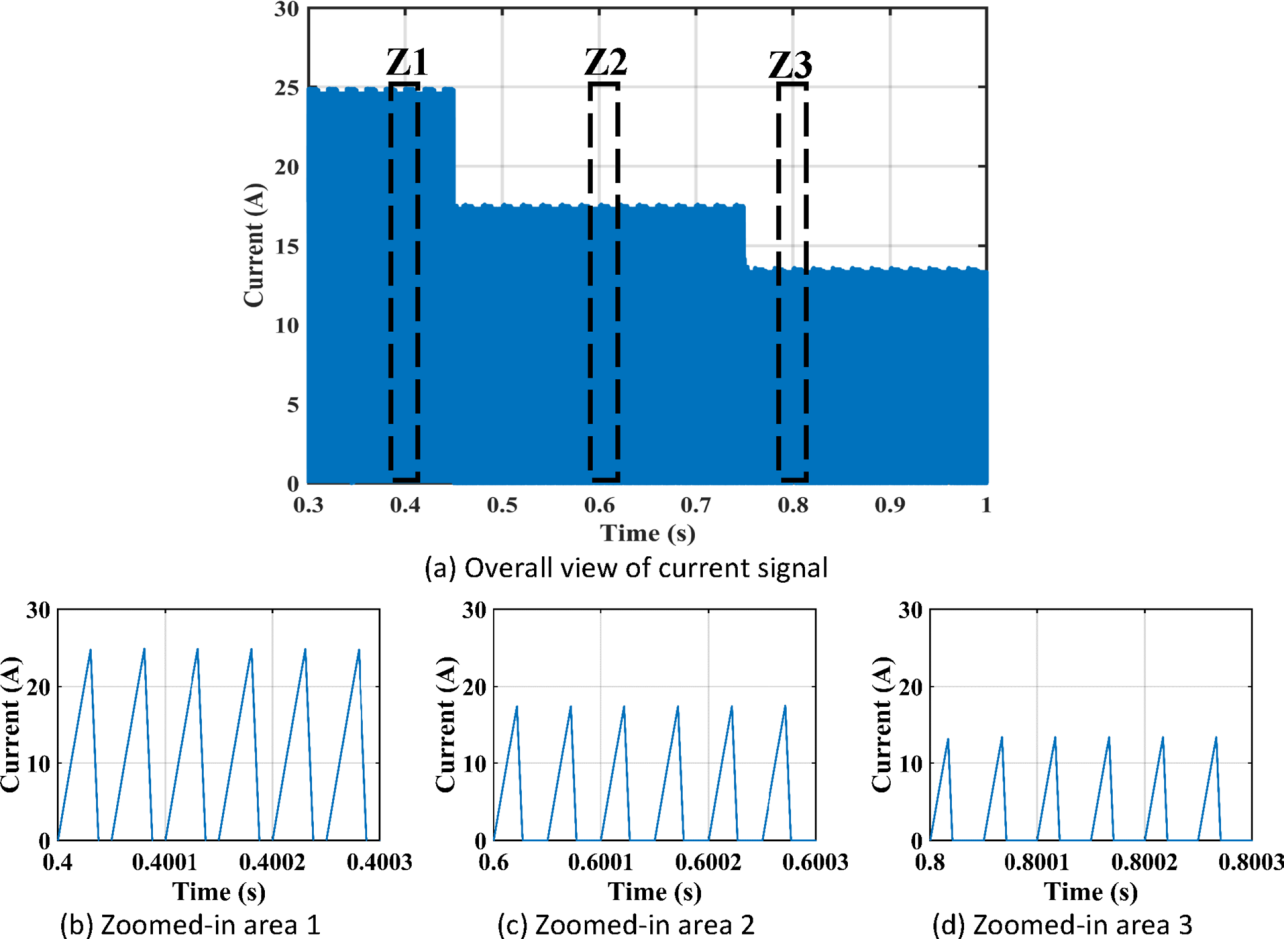
Response of $\:{i}_{{L}_{1}}\:$at different irradiance levels.

**Fig. 13:**
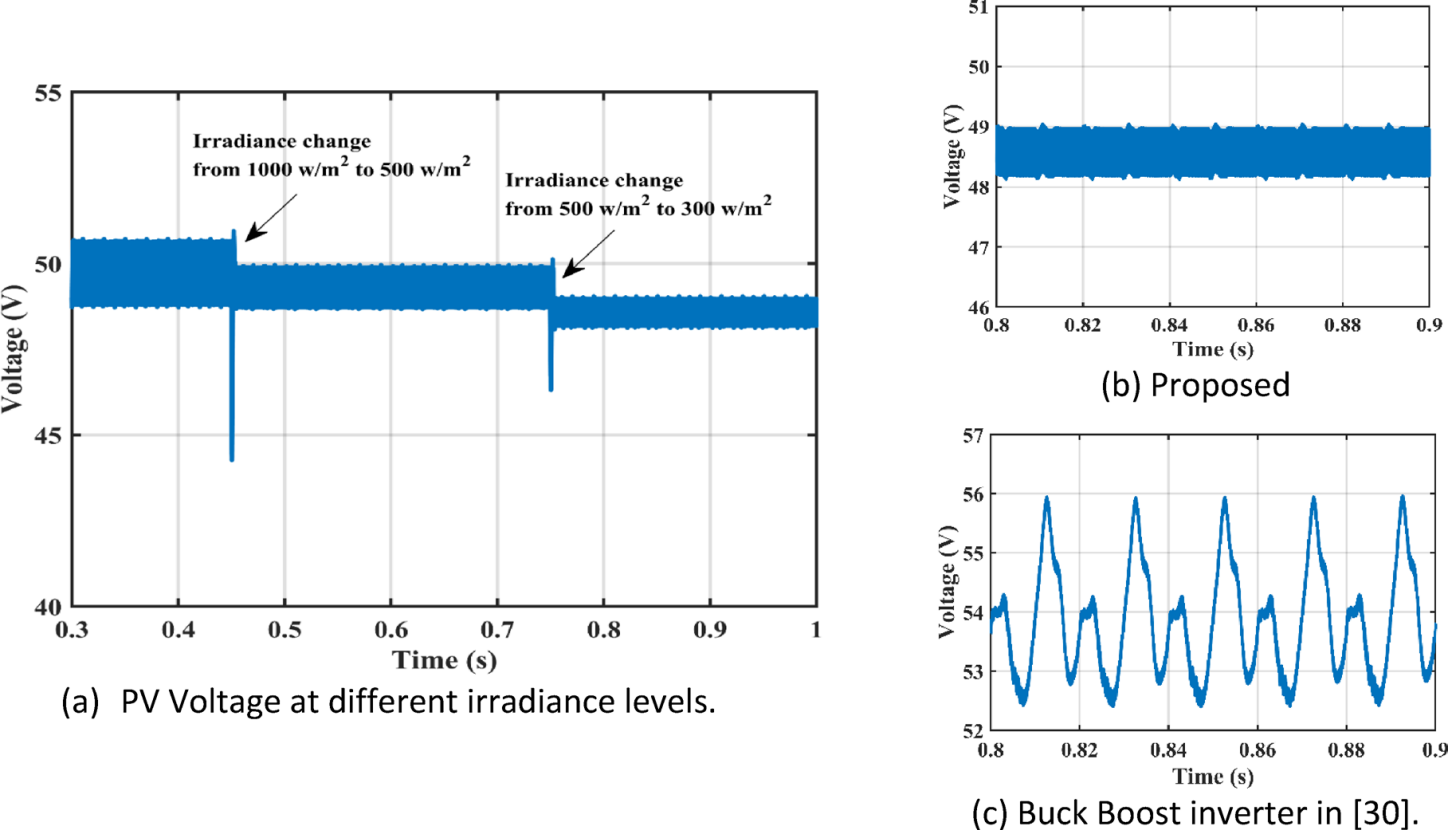
Low Frequency PV Voltage ripple difference.

**Fig. 14:**
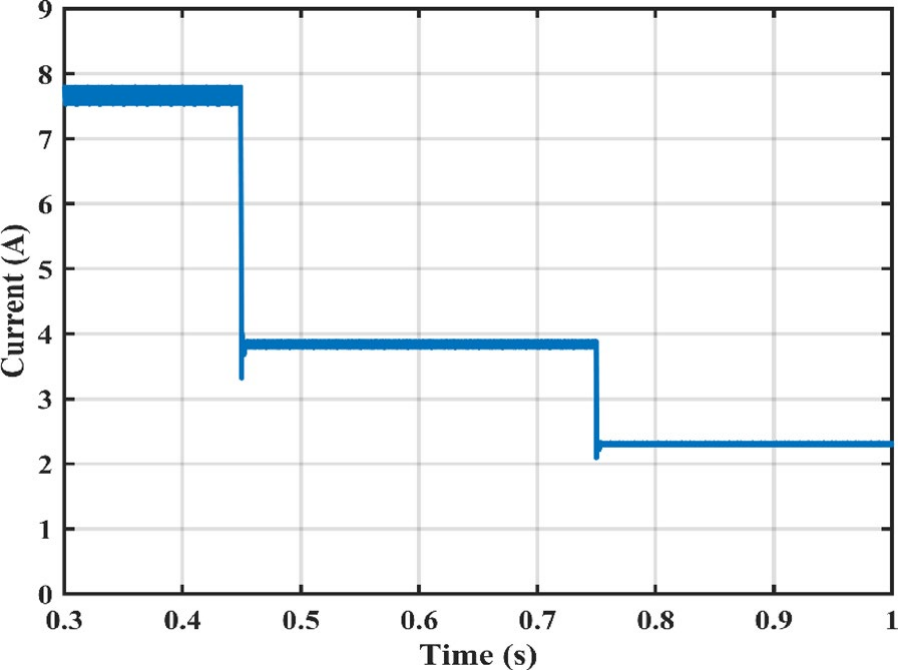
PV current at different irradiance levels.

Figure 11 shows the duty cycles of each independent stage. As seen from the figure the duty cycle $\:{D}_{1}$is less than $\:{D}_{2}$ which proves the independent operation of each converter regardless of the presence of a floating interval for the intermediate capacitor. The ripple in $\:{D}_{1}$ is negligible thanks to the DCM functionality of the first stage illustrated in Fig. 12 which decreases the 2nd order disturbance that flows to the PV module. This gives great flexibility for the design of voltage gain and enables higher efficiency and lower voltage burden on the semi-conductor devices. Figure 13 displays the appropriate PV voltage waveforms. The presented circuit has a negligible low frequency ripple if compared with the Buck-Boost converter proposed in^30^. Figure 14 shows the PV module current, and the simulation shows the efficiency of the designed control setup in terms of wide stability margin and faster response. The PV terminal voltage and power show no low-frequency ripple, indicating that the main objective has been achieved. At 1000 $\:w/{m}^{2}$, the PV module Voltage and current are 50 V and 7.65 A as dictated by the MPPT algorithm. And the output grid current is 2.9 A (RMS).

**Fig. 15:**
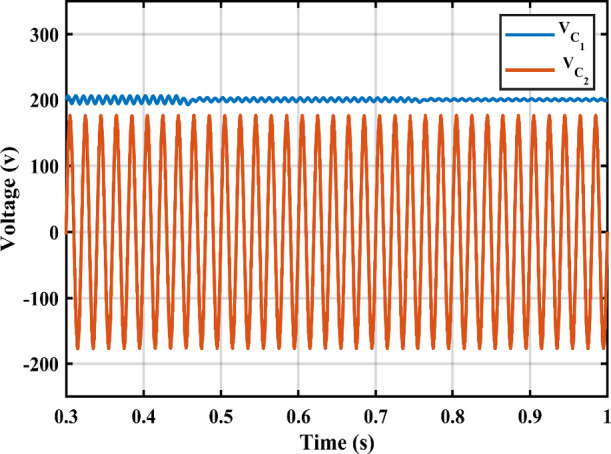
DC link capacitor voltage $\:{\:V}_{{C}_{1}}$and output capacitor voltage $\:{\:V}_{{C}_{2}}$.

**Fig. 16:**
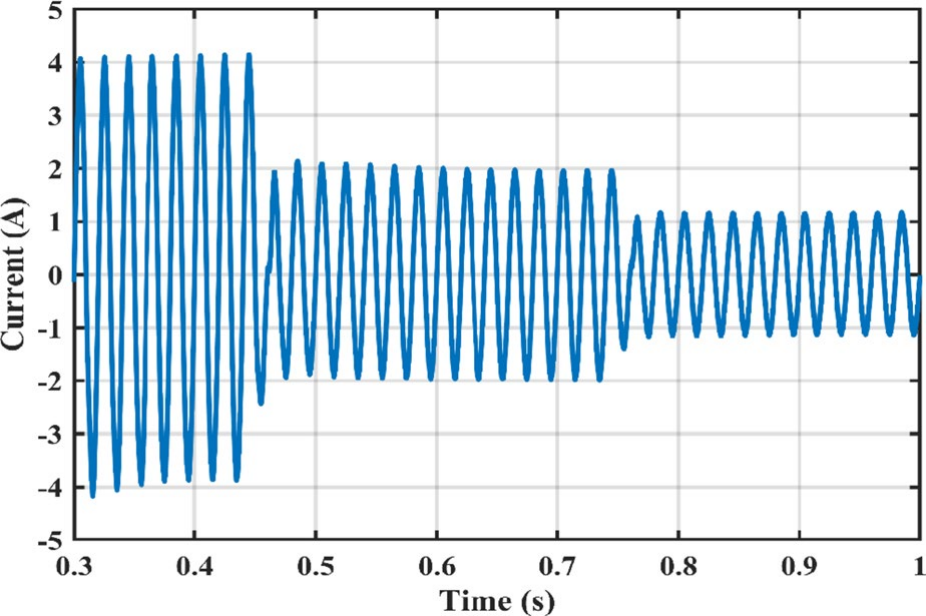
Grid currents $\:{I}_{g}$ at different irradiance levels.

**Fig. 17:**
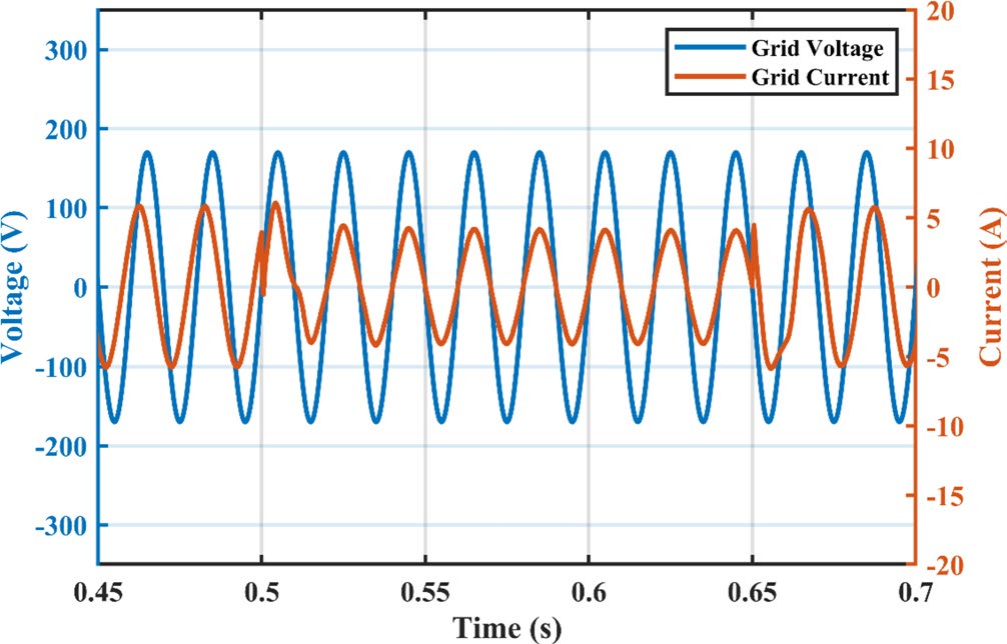
Grid voltage and currents at different power factor levels.

Irradiance drops to 500 $\:w/{m}^{2}$at $\:\text{t}=0.45\:$s. Vin shows negligible fluctuation, and $\:{I}_{pv}\:$falls to the appropriate MPP point of 3.9 A. The PV panel’s output voltage in Fig. 13 displays negligible ripple, indicating a fantastic utilization ratio of more than 98%. Figure 15 shows the plot for $\:{\:V}_{{C}_{1}}\left(t\right)\:$ where the DC link voltage pulses at 100 Hz, with a mean amount of 200 V. behavior of the circuit with a grid connected applications is shown in Fig. 16. Grid current is reported to decrease as irradiance levels drop and MPP algorithm switches to a lower output power setting. Again, at $\:t=0.75\:s$, the irradiance changes abruptly to 300 $\:w/{m}^{2}$. As a result, the PV current decreases quickly to a new level of 2.4 A and the grid current decreases to 0.75 A (RMS). The grid current controller shows an excellent transient response with approximately half a grid cycle to settle to a new value. Figure 15 shows a slight drop in the value of $\:{\:V}_{{C}_{1}}\left(t\right)\:$ at the irradiance level change instant. But the PI controller was able to reject the disturbance within 0.02 s which shows a good dynamic response and sufficient stability margin. The inverter system can also supply and absorb reactive power independent from active power generation as illustrated in Fig. 17. The Power factor (P.F) changes from unity to Leading to lagging at different time instants.

**Fig. 18:**
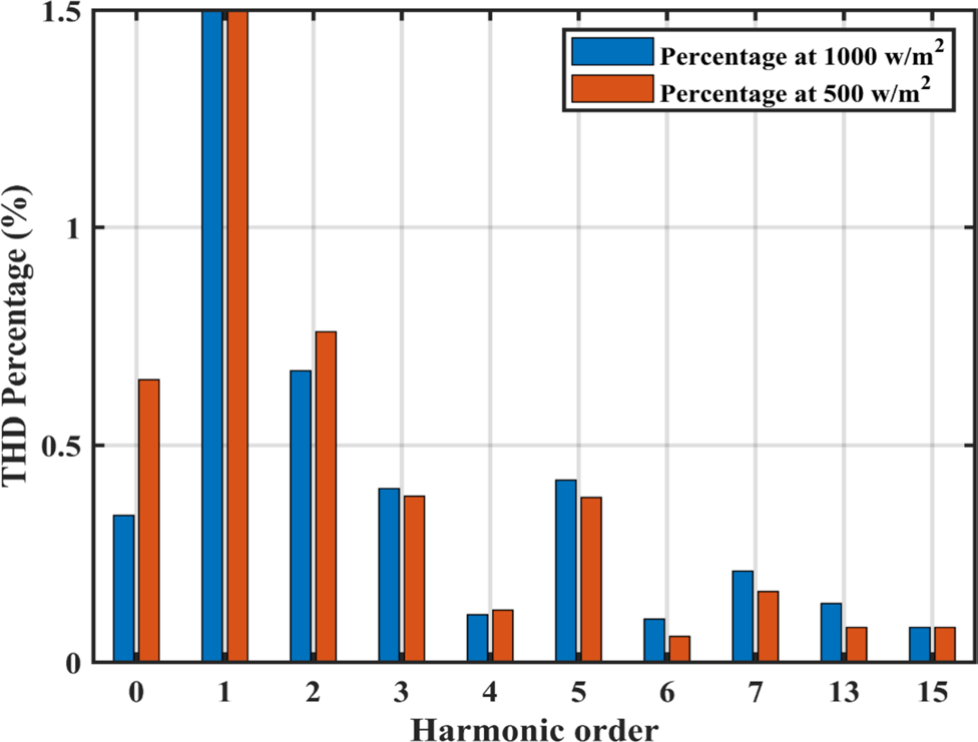
THD at different irradiance levels.

The negative terminal of the PV panel is linked to the grid, hence no common mode current exists. The grid current ($\:{i}_{g}$) has a THD of 1.56% (2.89 A fundamental). The grid current harmonic spectrum at 1000 /2 and 500 /2 is displayed in Fig. 18, where the highest harmonic component is less than 1.5%.

Compared to the topology proposed in^30^, the use of a lower DC-link voltage in the proposed design results in reduced voltage stress on the inverter stage switches ($\:{S}_{2}$ -$\:\:\:{S}_{5}$), while the current stress remains typical for a grid-tied inverter and is primarily dictated by the output load. The peak current through these switches can be estimated using the peak ripple value which can be derived easily from (20).

For the first-stage devices ($\:{S}_{1}$,$\:{\:S}_{6}$ and $\:{D}_{1}$,$\:{\:D}_{2}$), the maximum voltage stress occurs during distinct intervals of the switching cycle. However, each of these components experiences a maximum voltage approximately equal to half of the PV input voltage plus the DC-link voltage. The current stress for these components can be determined using (9). A summary of the maximum expected voltage and current stress values is provided in Table 3:

**Table 3:** Stress analysis.

Component	Maximum Voltage	Maximum Current	Values
$\:{S}_{1}$ &$\:\:{\:S}_{6}$	$\:\frac{{V}_{pv}+{{V}_{dc}}^{\text{*}}}{2}$	$\:\frac{{V}_{pv}{D}_{1}}{{f}_{sw}{L}_{1}}$	(125 V, 25 A)
$\:{D}_{1}\:$& $\:\:{D}_{2}$	$\:\frac{{V}_{pv}+{{V}_{dc}}^{\text{*}}}{2}$	$\:\frac{{V}_{pv}{D}_{1}}{{f}_{sw}{L}_{1}}$	(125 V, 25 A)
$\:{S}_{2}\:-\:\:{S}_{5}$	$\:{{V}_{dc}}^{\text{*}}$	$\:\frac{{{V}_{dc}}^{\text{*}}}{4{f}_{sw}{L}_{2}}$	(200 V, 6.3 A)

### Efficiency estimation

A simulation-based efficiency analysis was conducted to identify the major loss contributors in the proposed microinverter and to optimize component selection for improved performance. The simulations were performed using PLECS^33^ software, which offers fast simulation times and supports detailed manufacturer-based device models through lookup tables. These models allow accurate estimation of both conduction and switching losses for semiconductor devices. The primary sources of losses are summarized in Table 4:

**Table 4:** Major loss contributors.

Component	Major Loss Contribution
$\:{S}_{1}$ &$\:\:{\:S}_{6}$	Conduction & Turn off losses
$\:{D}_{1}\:$& $\:\:{D}_{2}$	Conduction Losses only
$\:{S}_{2}\:-\:\:{S}_{5}$	Conduction & Switching losses
$\:{r}_{1}$	Conduction losses
$\:{R}_{damping}$	Conduction losses

As shown in the table, switches $\:{S}_{1}$ and $\:{S}_{6}$ operate in discontinuous conduction mode (DCM), which results in minimal turn-on losses and moderate turn-off losses. This operational mode also benefits diodes $\:{D}_{1}$ and $\:{D}_{2}$, as they experience zero current switching (ZCS) during turn-off, effectively eliminating reverse recovery losses. The inverter-stage switches ($\:{S}_{2}$ -$\:\:\:{S}_{5}$) are subject to both conduction and switching losses, while conduction losses from passive components such as the input inductor resistance ($\:{r}_{1}$) and damping resistor also contribute to the overall losses.

**Fig. 19:**
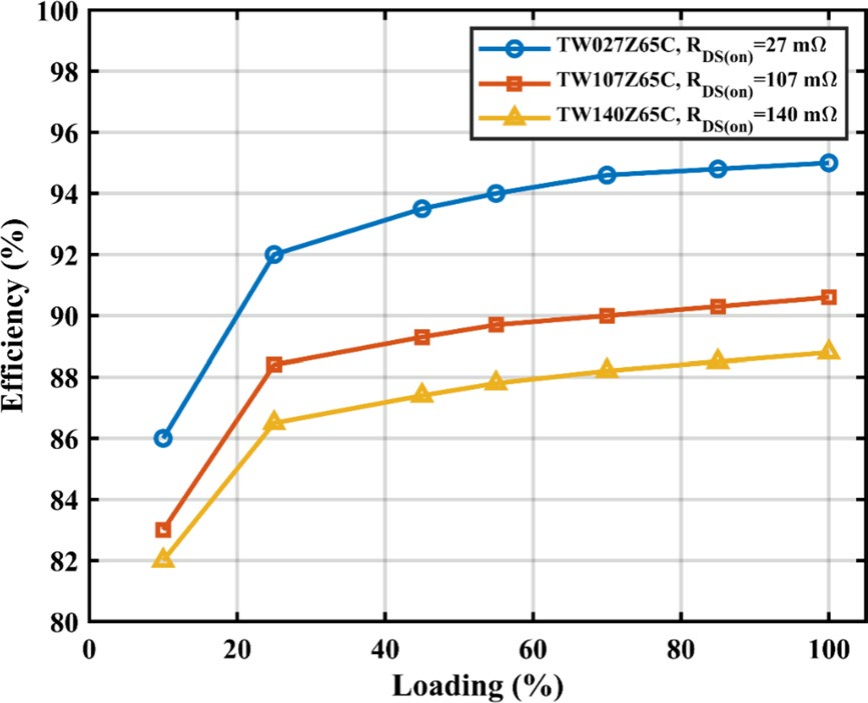
Efficiency vs. loading for different switch models.

Using the circuit parameters provided in Table II, the simulation results show that switching losses are relatively small compared to conduction losses, primarily due to the low DC-link voltage and moderate switching frequency. This design choice results in high efficiency over a wide load range. However, conduction losses—particularly from $\:{S}_{1}$ and $\:{S}_{6}$—still significantly impact efficiency. Therefore, selecting low $\:{R}_{\text{D}\text{S}\left(\text{o}\text{n}\right)}$ MOSFETs is critical to maintaining high efficiency while minimizing thermal and size constraints. To support this, Fig. 19 presents a simulation plot of efficiency versus loading percentage, comparing three different MOSFETs with varying on-resistance values. The results clearly demonstrate that the proposed circuit can achieve efficiencies as high as 95%, provided that conduction losses are minimized—especially in $\:{S}_{1}$ and $\:{S}_{6}$—through optimal device selection.

### Standalone system

The Topology proposed in this paper can also support standalone applications feeding power to isolated loads requiring different voltage and power levels. The values shown in Table V are utilized for the simulation study as well as the fabricated prototype. This part’s purpose is to be a reference for assessment of the practical results against the theoretical work.

**Table 5:** Prototype Parameters.

Parameters	Values
$\:{C}_{pv}$	100 µF
$\:{L}_{1\:},\:{r}_{1}$	40 µH, 200 mΩ
$\:{C}_{1}$	330 µF
$\:{L}_{2},\:{\:r}_{2}$	1 mH, 500 mΩ
$\:{C}_{2}$	10 µF
$\:{R}_{load}$	60 Ω
$\:{f}_{sw}$	11 KHz
$\:{{V}_{dc}}^{\text{*}}$	100 V
PV Panel	Kyocera KD40 series

**Fig. 20:**
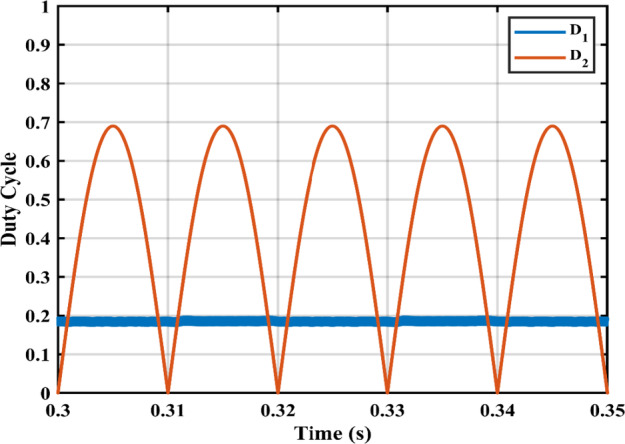
Duty ratio variations for $\:{D}_{1}$ and$\:{\:D}_{2}$.

**Fig. 21:**
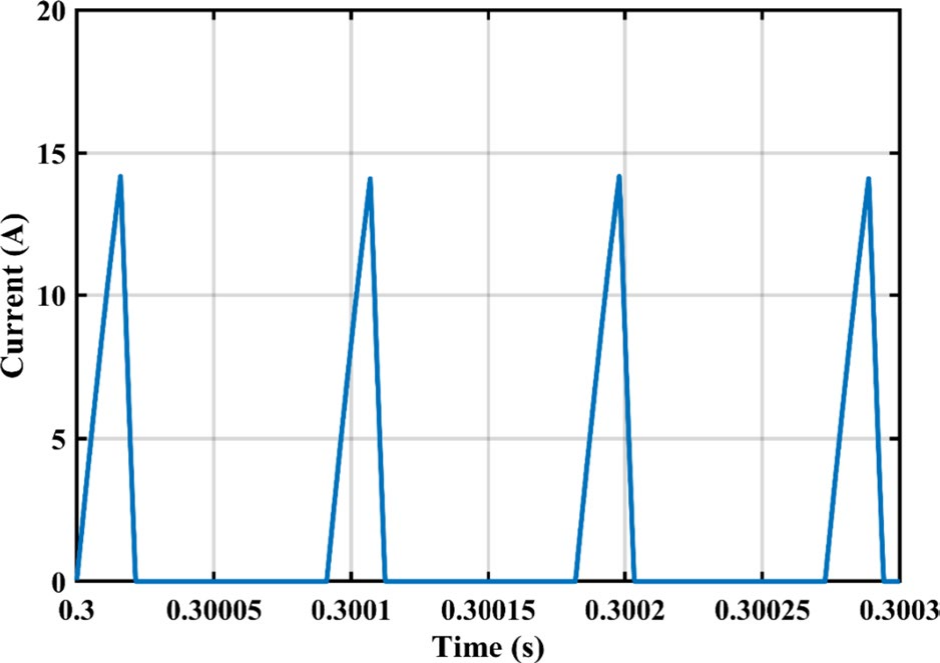
DCM Current for $\:{L}_{1\:}$.

**Fig. 22:**
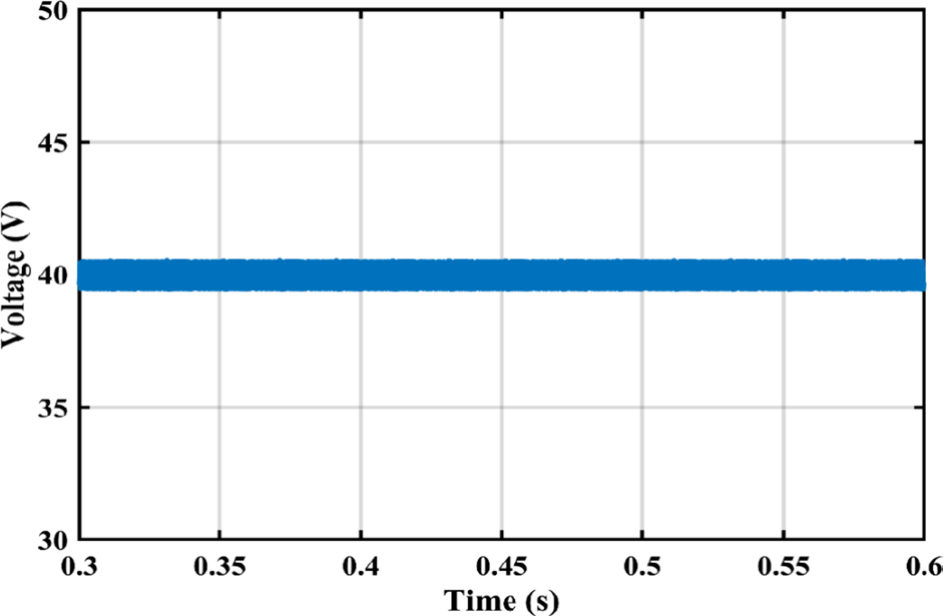
Illustration of PV panel voltage curve.

**Fig. 23:**
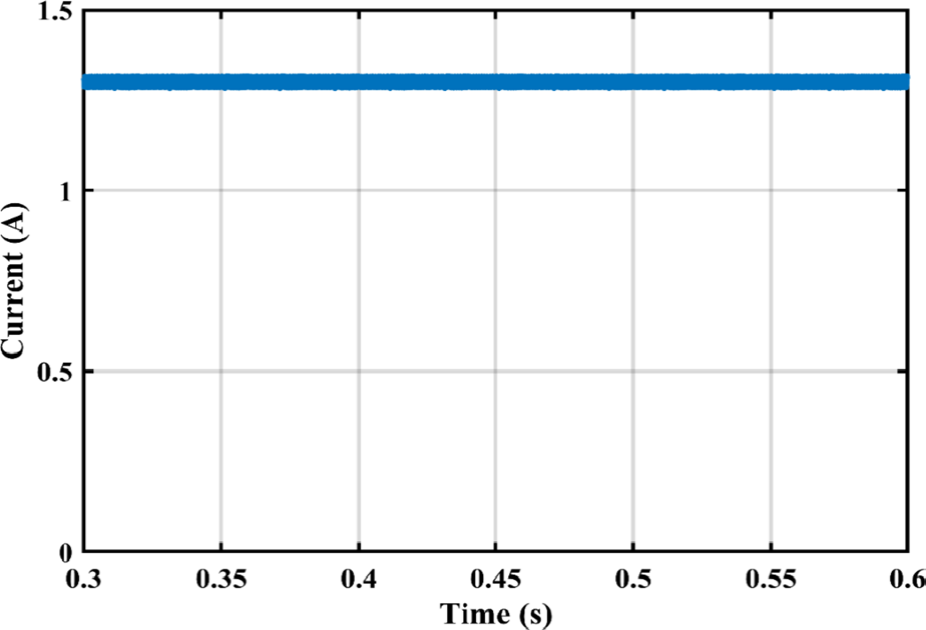
Illustration of PV panel current curve.

**Fig. 24:**
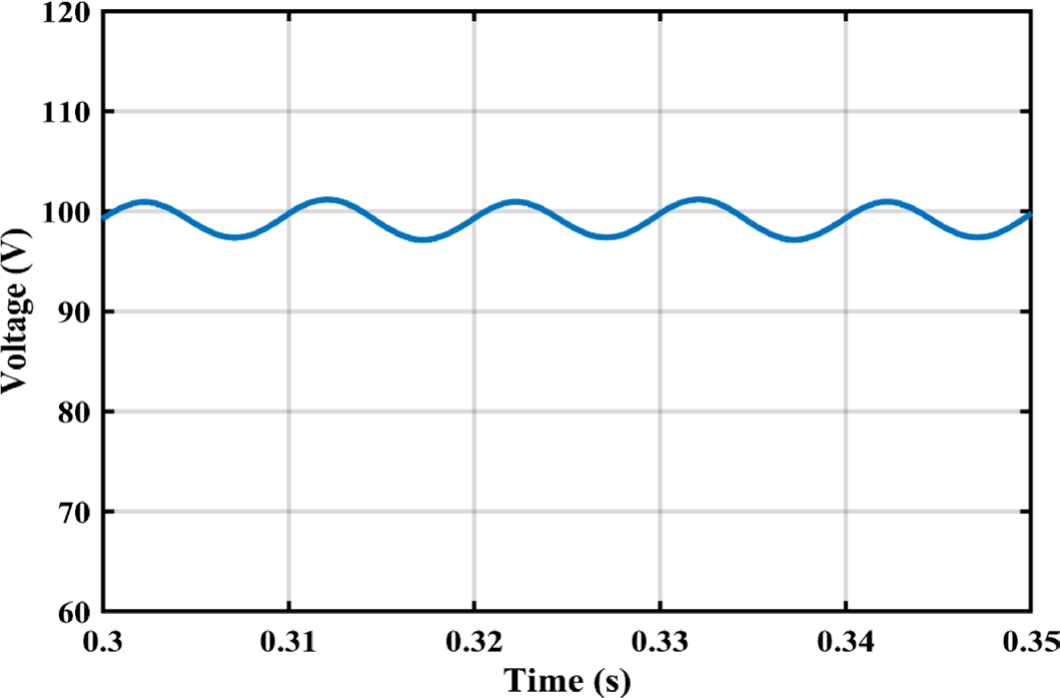
intermediate capacitor voltage $\:{V}_{{C}_{1}}$ waveform.

**Fig. 25:**
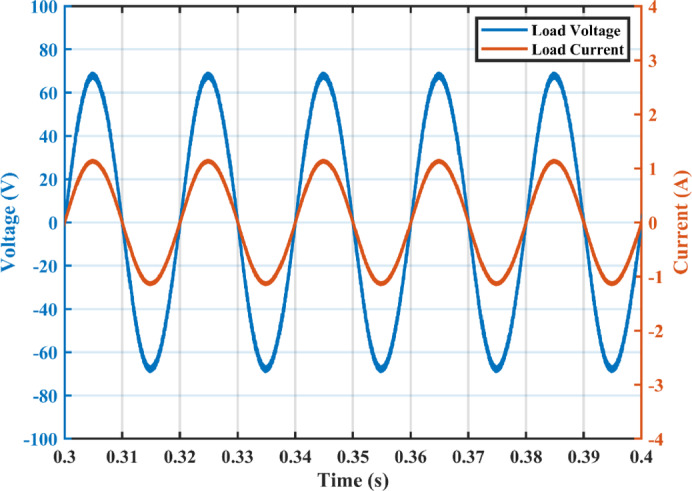
Load voltage and current waveforms.

The practical system is simulated at a constant irradiance level and the final values for the voltages, currents and controller duty cycle outputs are recorded. At maximum power conditions $\:{D}_{1}$and $\:{D}_{2}$ are illustrated in Fig. 20. As presented in Fig. 21, the inductor current $\:{L}_{1\:}$is discontinuous to provide total decoupling between the input and output stages. The PV panel used for the simulation and fabricated prototype outputs 40 V and 1.3 A at optimum conditions. The waveforms for the voltage and the currents are shown at steady state values in Figs. 22 and 23 respectively. No 2nd harmonic ripple is present in the DC quantitates as per design requirements. The intermediate capacitor voltage is regulated at a constant average value of 100 V as shown in Fig. 24 this enables the inverter stage to output a suitable AC voltage. Lastly, the inverter output waveforms are illustrated in Fig. 25. 60 Ω is the load the inverter supplies. The estimated RMS values for the voltage and the current are 50 V and 0.84 A respectively.

## Experimental validation

**Fig. 26:**
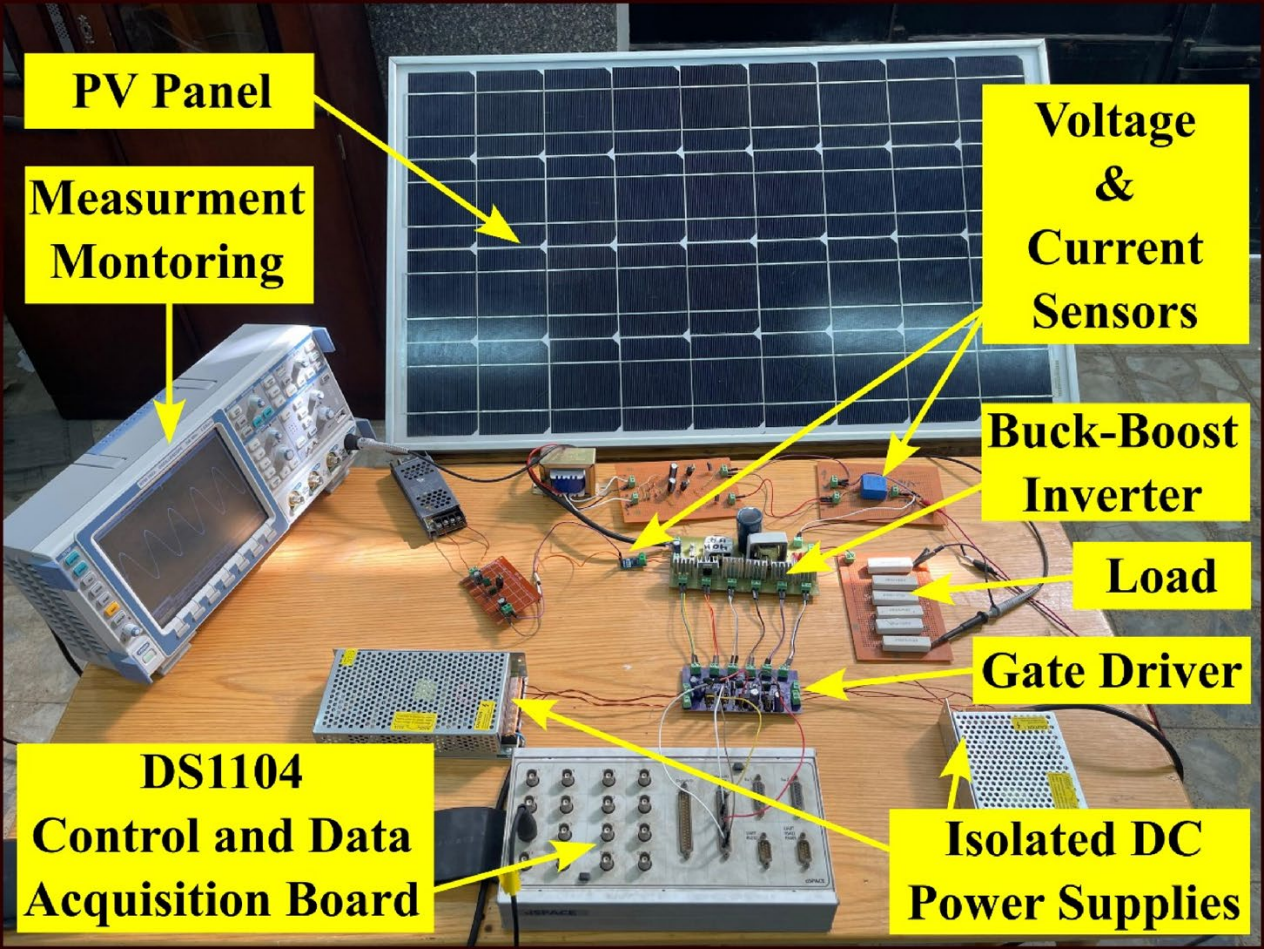
Experimental setup.

A 50 W testing model is built, and extensive tests are performed to verify the viability of the suggested circuit. The model makes use of the parameters listed in Table 5 as mentioned before. Table 6 provides the details for the equipment that was utilized. Figure 26 shows a picture of the experimental configuration.

**Table 6:** Laboratory prototype Components.

Parameters	Values
$\:{S}_{1}-{S}_{6}$	IRF740 N-Channel MOSFET
$\:{D}_{1}-{D}_{2}$	BYW98-200
Voltage sensor	LV25-P
Current sensor	ACS712
Gate Driver	TLP250
DSP	DS1104 R&D Controller Board

**Fig. 27:**
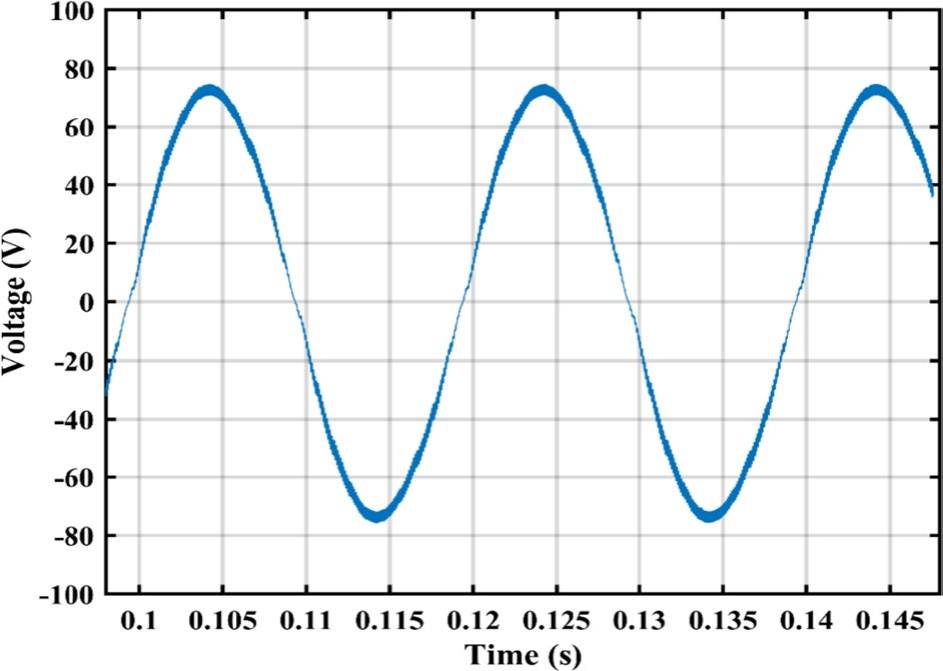
Output voltage.

**Fig. 28:**
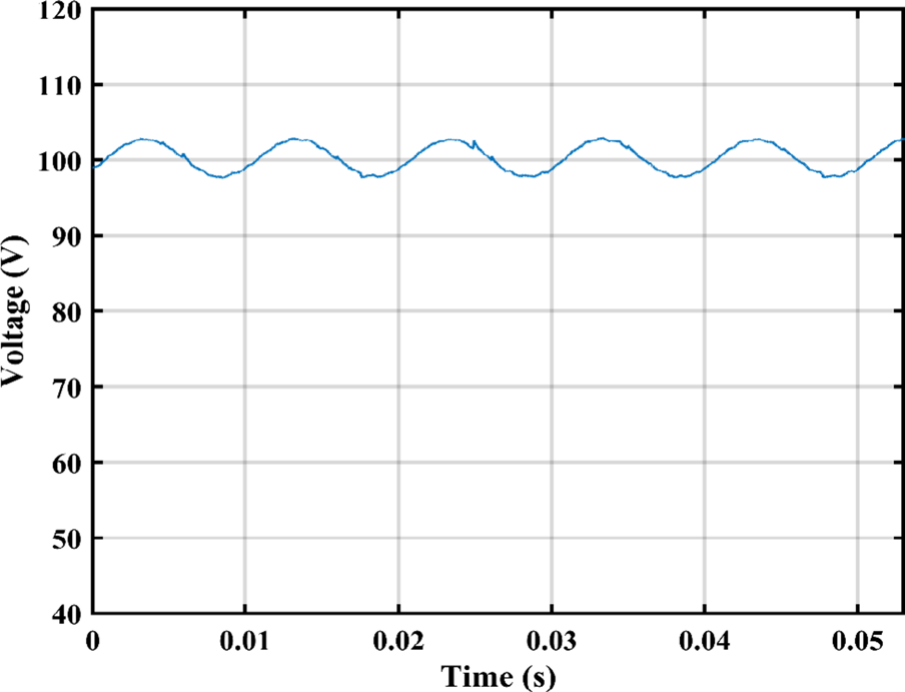
DC link voltage.

The proposed circuit has an PV input voltage of 40 V and its output terminals are coupled to a load of 60 Ω. The measured voltage is displayed in Fig. 27, The inverter’s output voltage approximately equals 50 VRMS and The mean input current is 1.3 A. The average voltage across $\:{C}_{1}\:$is maintained at 100 V as shown in Fig. 28. Second order harmonic ripple of approximately 5 V peak to peak can be observed in $\:{V}_{{C}_{1}}$, This attests to the capacitor’s function as a buffer between the DC and AC sides.

**Fig. 29:**
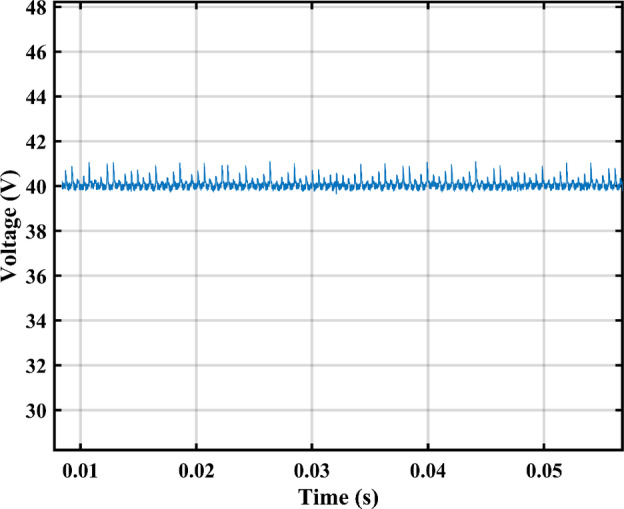
Input voltage for the PV panel.

**Fig. 30:**
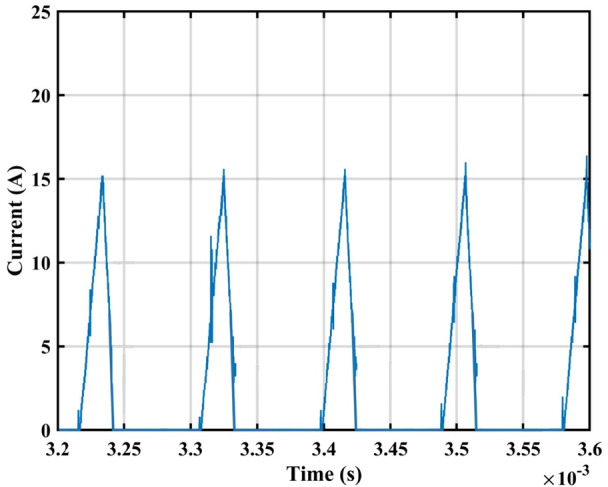
inductor $\:{L}_{1\:}$ DCM Current.

**Fig. 31:**
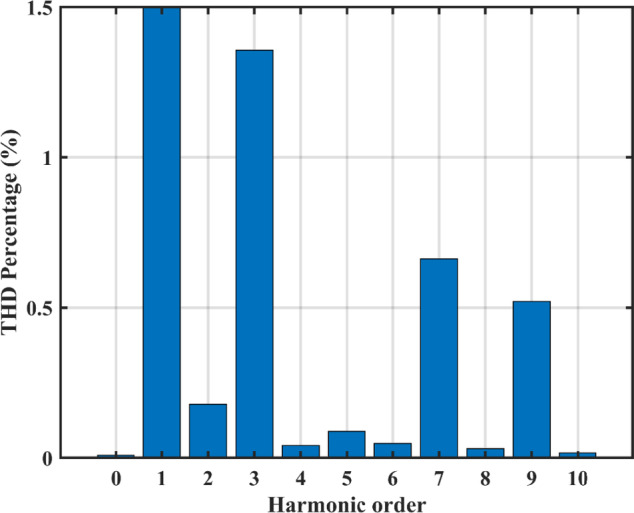
THD of the measured output voltage.

On the other hand, almost no ripple exists on the DC side as in Fig. 29. By investigating the waveform of inductor current $\:{L}_{1}$, we clearly see the DCM operation in Fig. 30, during the charging phase the current reaches a peak of approximately 15 A which closely agrees the simulation results. The controller output duty cycle for $\:{D}_{1}$ and $\:{D}_{2}$ are 0.17 and 0.76 respectively, this further proves the unconstrained operation of the topology allowing it to function with a broad spectrum of voltages and currents. The measured voltage spectrum is shown in Fig. 31 where each component is less than 1.5% this amounts to a total THD of 2.3%.

Despite the use of a 50 W prototype for proof-of-concept validation, the proposed circuit is inherently scalable to higher power levels by appropriately resizing passive components and selecting semiconductor devices with suitable ratings. This scalability is demonstrated in the “Simulation Results” section, where a nearly 400 W grid-connected system—modelled with different system parameters—was simulated. The results confirmed consistent performance and control behaviour, validating the robustness and scalability of the proposed topology for practical residential microinverter applications. Furthermore, the topology eliminates bulky electrolytic capacitors and minimizes common-mode currents, both of which contribute to improved reliability, thermal performance, and electromagnetic compatibility (EMC)—key requirements for high-power applications.

## Comparative study

In this section several attributes of the suggested circuit are assessed against the buck boost inverter proposed in^30^ to identify the various merits and the challenges associated with each topology. While the proposed inverter topology employs a slightly higher number of semiconductor devices—specifically, six MOSFETs and two diodes—compared to some alternative designs such as^30^, which uses around five switches, this modest increase is a calculated trade-off that results in clear system-level benefits.

Firstly, by enabling discontinuous conduction mode (DCM) operation in the first stage and constant gain for the inverter stage, the topology supports a simplified and predictable control scheme. This significantly reduces computational complexity and allows for the use of cost-effective microcontrollers with lower processing capabilities, rather than relying on high-performance digital signal processors (DSPs) or FPGAs.

Secondly, despite the higher device count, the individual semiconductor devices operate under lower voltage and current stress, enabling the use of less expensive, lower-rated components—offsetting the added cost due to increased quantity.

Finally, the topology’s ability to support a wide PV input voltage range eliminates the need for an additional front-end DC-DC conversion stage. This contributes to a simpler and more compact system design, while reducing total system cost, improving reliability, and enhancing efficiency. Table 7 summarizes the most important features for both circuits.

**Table 7:** Assessment of the suggested circuit with topology proposed in^30^.

Point of comparison	Proposed topology	Buck boost inverter proposed in^30^
Number of Components	6 Switches, 2 inductors, 2 Capacitors, 2 Diodes.	5 Switches, 2 inductors, 2 Capacitors.
Switching Frequency	20 KHz Every half cycle, 2 switches run at low frequency and 4 switches operate at high frequency.	50 KHz All 5 switches operate at High frequency.
Power Decoupling	Inherent	Inherent
PV Voltage and current ripple	Negligible ripple	More ripple ($\:\approx\:3.5\:V)$
Dual Grounding	yes	Yes
Operation	DCM for 1 st stage & CCM for the 2nd stage	CCM
Inverter gain	Constant gain Reduces controller complexity and design	Variable gain, changes every half cycle More constrained design and complexity for the controller.
Grid Voltage interface	110 V	110 V
DC Link Voltage	200 V	250 V
Voltage stress	Less Voltage stress (up to 250 V)	More Voltage stress (up to 300 V)
Range of operation	40–270 V	40–100 V
Reactive Power injection control	yes	yes
Constraints	1. $\:{V}_{c1}>{V}_{grid}$	1. $\:{V}_{c1}>{V}_{grid}$ 2. $\:\frac{{V}_{c1}}{{V}_{c1}+{V}_{PV}}>\frac{{V}_{grid}}{{V}_{c1}}$ 3. $\:\left(1-{D}_{1max}-{D}_{2}\right){T}_{s}\frac{{V}_{c1}}{{L}_{1}}>{I}_{2}+{D}_{1max}{T}_{s}\frac{{V}_{c1}+{V}_{PV}}{2{L}_{2}}$

## Conclusions

This work proposes an original single-phase buck boost grid-connected transformer-free microinverter circuit. The presented circuit uses 6 switches, 2 of which run on grid frequency every half cycle while the other 4 runs at high frequency. For optimal performance, the described microinverter is connected to a 110 V AC supply. It can support standalone as well as grid connected applications. Since the microinverter already can provide power decoupling, active power decoupling technique or big capacitor is not required. The inverter’s reliability is increased because all of the capacitors are thin film types. And since a common connection between the PV panel and the grid exists, the leakage current’s magnitude is zero. The DCM’s buck-boost stage reduces the inductors’ bulk while guaranteeing a greater voltage amplification factor and minimal switching losses. In order to achieve low THD and reactive power injection capabilities, the Buck stage is operated in CCM. The microinverter requires no floating interval for proper operation. To ensure power decoupling capability and thus has a wider range of operation.

The proposed microinverter has been subjected to studies, and design recommendations for the passive components are also given. Results from simulations are used to define and validate the suggested power decoupling technique. The system’s dynamic analysis, operational range, and controller design have all been thoroughly covered. A simple model was developed and implemented to verify the results.

## Data Availability

The data that support the findings of this study are available from the author, Y.N.A., upon reasonable request.
